# Mobile-Phase Contributions to Analyte Retention and Selectivity in Reversed-Phase Liquid Chromatography: 1. General Effects

**DOI:** 10.1021/acs.jpcb.5c01695

**Published:** 2025-06-13

**Authors:** Andreas Steinhoff, Alexandra Höltzel, Ulrich Tallarek

**Affiliations:** Department of Chemistry, 9377 Philipps-Universität Marburg, Hans-Meerwein-Strasse 4, Marburg 35032, Germany

## Abstract

Analyte retention in reversed-phase liquid chromatography is manipulated via the elution strength of the water–organic solvent (W–OS) mobile phase, whereby raising the OS volume fraction or eluent strength (methanol < acetonitrile) lowers retention. We investigated this effect at the molecular level through molecular dynamics simulations in a slit-pore model of a C_18_ stationary phase, using the solute benzene to trace the composition and occupation of the immediate analyte environments involved in solute partitioning into the bonded-phase region and solute adsorption to the interfacial region. Spatially resolved contact analysis revealed that the number of bonded-phase contacts per analyte molecule decreases from the bonded-phase region over the extension of the interfacial region while the number of solvent contacts increases. The analyte density distribution in the stationary phase is sensitive to the local W density, which is controlled by the mobile-phase parameters. With increasing mobile-phase elution strength, the W density recedes from the interfacial region, favoring the occupation of analyte environments closer to the bulk liquid region. The ensuing redistribution of analyte density within the stationary phase results in an overall loss of bonded-phase contacts, tantamount to loss of retention. The retentivity of the stationary phase therefore depends on its solvation by the mobile phase.

## Graphical abstract

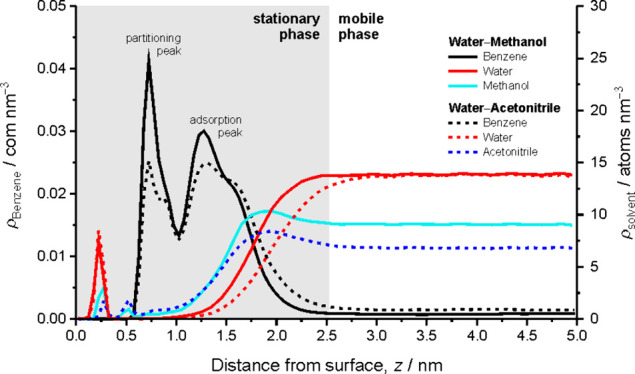

## 1 Introduction

In high-performance liquid chromatography (HPLC) practice, the majority of small molecule separations is carried out in reversed-phase liquid chromatography (RPLC) mode on silica-based columns. The column is selected first, as its properties, particularly its stationary-phase chemistry, determine the analyte spectrum to a large extent.^1^ The stationary phase consists of the solid silica surface and the attached bonded phase, which is either fully or predominantly hydrophobic (e.g., alkyl or polar-embedded phases, respectively).^2,3^ The water–organic solvent (W–OS) mobile phase is chosen for its compatibility with the selected column, the sample, and the detector.^4^ The ancillary choice of the mobile phase belies its importance for the separation. Beyond the solvation and transport of analyte molecules through the pore space of the column, the functional role of the mobile phase comprises the solvation of the stationary phase and the control of analyte retention.^5^

Analyte retention is quantified by the phase-based retention factor *k*, which is related to the distribution coefficient *K* through the column-specific phase ratio β according to

$$k=K\beta =\frac{c_{\mathrm{SP}}}{c_{\mathrm{MP}}}\frac{V_{\mathrm{SP}}}{V_{\mathrm{MP}}}$$ 1

where *c*_SP_ and *c*_MP_ are the analyte concentrations in the solvated stationary phase and the bulk liquid mobile phase, respectively, and *V*_SP_ and *V*_MP_ are the volumes occupied by the stationary phase (solid silica support with attached bonded phase) and the mobile phase in the column.^6^ Equation 1 reflects that the retention factor decreases when analyte concentration is shifted from the stationary-phase compartment to the mobile-phase compartment of a column. In practice, analyte retention is manipulated through the elution strength of the W–OS mobile phase, which has two variables: the volume fraction and the eluent strength (chemical identity) of the OS.^7^ Increasing the OS volume fraction in the mobile phase decreases analyte retention in general, but rarely changes the order in which different compounds elute from the column. Increasing the OS eluent strength, and thus changing the chemical composition of the mobile phase, may additionally alter the retention of two analytes relative to each other, referred to as selectivity α = *k*_2_/*k*_1_ (whereby *k*_2_ ≥ *k*_1_ per definition). The outcome of an OS change on a separation is difficult to predict, because the mobile-phase contributions to analyte retention and selectivity remain poorly understood.^8,9^

The mobile-phase elution strength is a purely empirical concept. The assignment of weak and strong solvent to the components of a binary mixture as well as the ranking of solvents according to their eluent strength relies on the observed elution behavior in a given HPLC mode and is rationalized by perceived similarity to the stationary-phase chemistry. For example, the OS is the strong eluent of the W–OS mobile phase in RPLC with hydrophobic stationary phases, whereas W is the strong eluent in hydrophilic interaction liquid chromatography (HILIC) with hydrophilic stationary phases. Raising the volume fraction of the strong eluent in the mobile phase is supposed to decrease analyte retention by reducing the hydrophobicity or hydrophilicity gradient to the stationary phase (RPLC or HILIC, respectively).

Chemical similarity arguments cannot be extended to the eluent strength, which is conceived as the effectiveness of a given solvent in displacing analyte molecules from the stationary phase. In RPLC, methanol (MeOH) has lower eluent strength than acetone, tetrahydrofuran, or acetonitrile (ACN) when compared at equal volume fraction. Although approaches to quantify the OS eluent strength in RPLC have been in use for decades and continue to be developed,^7,8,10^ these do not explain why a particular solvent has higher or lower eluent strength than another solvent. What constitutes the eluent strength of a solvent has not been defined yet.

Although the definition of the retention factor (cf. eq 1) recalls solute partitioning between two immiscible liquids, the distribution of analyte density between the stationary-phase and the mobile-phase compartment of a column is more complex than liquid–liquid partitioning. The analyte adsorption isotherms of small compounds on C_18_ columns, acquired by frontal analysis measurements, reflect that the solvated stationary phase constitutes a heterogeneous environment to solute molecules.^11^ Additionally, molecular simulations of solute partitioning between liquid *n*-hexadecane and W–MeOH mixtures of varying MeOH content have shown that the solute distribution coefficient is sensitive to the extent of solvent penetration into the *n*-hexadecane phase, allowing the conclusion that the solvation of the hydrophobic stationary phase by the running mobile phase plays a role in analyte retention.^12^

A physicochemical explanation for the mobile-phase elution strength requires a molecular-level view on analyte retention in liquid chromatography.^13^ Building on knowledge about the influence of the mobile-phase composition on RPLC and HILIC separations gained from earlier molecular simulation studies,^14–17^ we recently began a molecular dynamics (MD) research program dedicated to establish a physicochemical framework for the contribution of the mobile phase to the interfacial phenomena that govern mass transport in RPLC.^18,19^ The parameters of the program were informed by standard conditions for small molecule separations in RPLC practice, which are carried out on silica-based, endcapped, dimethyl-*n*-octadecylsilane (C_18_) columns with W–MeOH or W–ACN mobile phases containing between 10 and 90 vol % OS. Our analyte ensemble consists of six, structurally closely related, aromatic hydrocarbon compounds, which elute from a C_18_ column in order of decreasing solute polarity as predicted by the logarithm of the *n*-octanol–W partition coefficient, log*K*_OW_.^20^ (Low, medium, and high solute polarity correspond to high, low, and negative values of log*K*_OW_, respectively.) Naphthalene, ethylbenzene, and benzene have low solute polarity (apolar analytes) and are thus more retained (i.e., elute later from the column) than acetophenone, benzyl alcohol, and phenol (moderately polar analytes).^6,19^

Our research program investigates the three successive steps of chromatographic separations, namely (i) column equilibration with the mobile phase, (ii) sample solvation in the mobile phase, and (iii) sequential elution of the injected sample components from the column with the mobile phase, through individual MD simulation studies where the chromatographic interface is represented by an RPLC slit-pore model.

Slit-pore models have been instrumental in establishing the existing molecular-level knowledge about the RPLC interface during the last 25 years.^13,15,21–26^ The slit-pore model approximates the solid–liquid interface inside a column by eliminating the surface curvature of the silica support (and thus the complex, macro–mesoporous column morphology) and reducing the silica surface chemistry to an idealized version. Simulations in cylindrical pore models have shown that the extent of surface curvature and the associated changes in surface chemistry can modify the local density and diffusivity of bonded-phase groups, solvent, and analyte molecules considerably.^26–29^ Yet, the empirical knowledge from RPLC practice has been very well recovered by simulations in slit-pore models,^18,23–26,30–32^ validating their use to represent the column-averaged properties of the RPLC interface, as recently confirmed by multiscale simulations of diffusive analyte transport in reconstructed macro–mesoporous column morphologies.^33^

The characteristic properties of the RPLC interface are shown in Figure 1, which introduces our RPLC slit-pore model with a snapshot from the simulation of the analyte benzene and the derived bonded-phase, solvent, and analyte density profiles. The bonded-phase region is characterized by high bonded-phase and low solvent density. Solvent presence in the bonded-phase region is predominantly (directly or indirectly) related to the coordination of residual OH groups at the silica surface. W density in the bonded-phase region is confined to the surface-adsorbed solvent layer, whereas OS density is present, if low, throughout the bonded-phase region. The bonded-phase density declines over the extension of the interfacial region while the solvent density increases. The W density increases from practically zero toward its respective density in the bulk liquid region, whereas the OS density starts from a low level and goes through a maximum in the interfacial region before reaching constant density in the bulk liquid region. The enrichment of OS molecules in the interfacial region (also referred to as the OS ditch^34^) is a distinctive feature of the RPLC interface and has been observed consistently in molecular simulations of C_18_ stationary phases^13,15,18,22,25–37^ and macroscopically as positive excess in the OS surface excess adsorption isotherms of C_18_ columns.^38^

**Figure 1:**
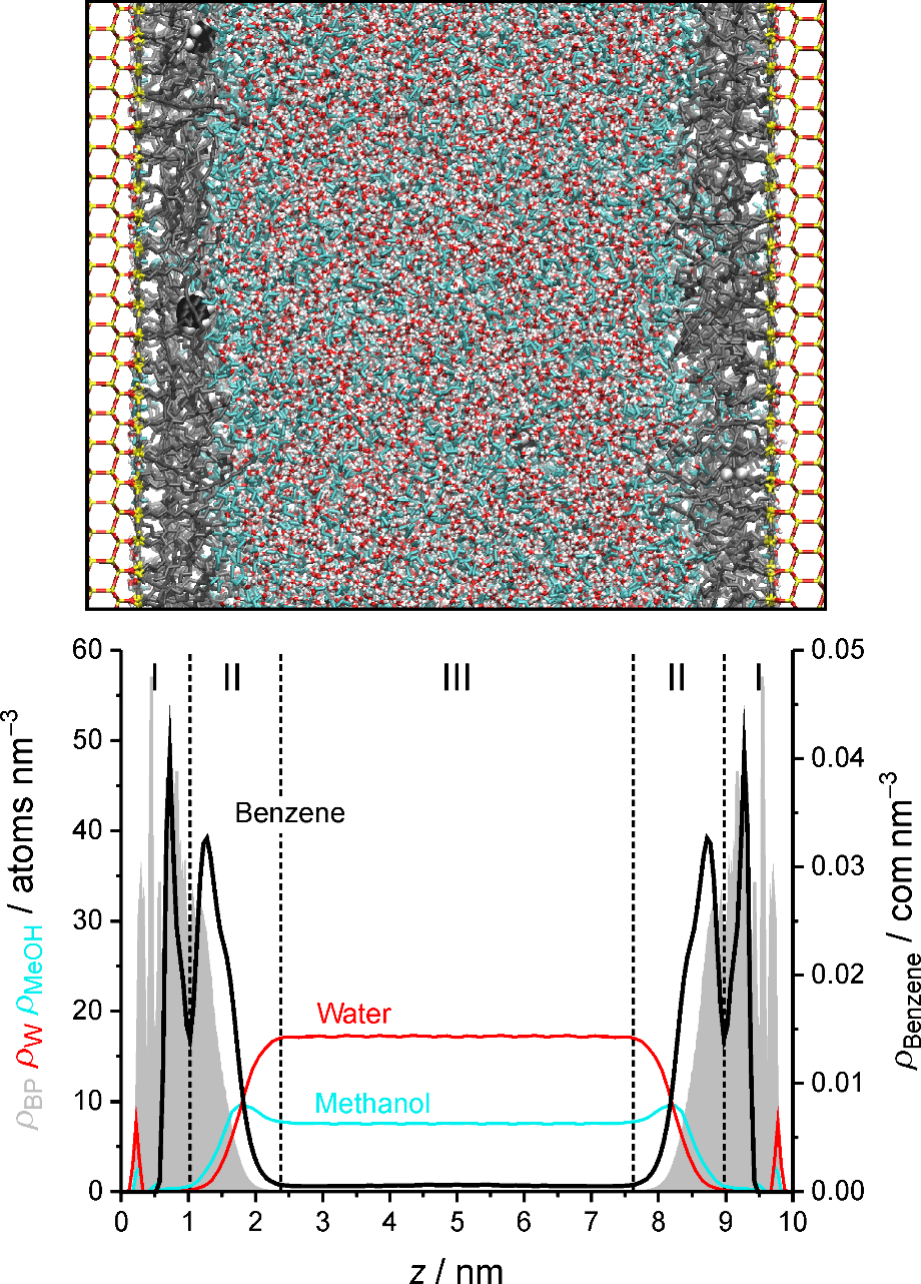
Top: Snapshot from the simulation of the analyte benzene in our RPLC slit-pore model with a W–OS mobile phase containing 50 vol % MeOH. Silica atoms (Si, O, and H atoms) are shown as yellow, red, and white sticks, respectively, and bonded-phase groups as gray sticks. O and H atoms of W molecules are shown as red and white sticks, respectively, and MeOH molecules as cyan sticks for visual discrimination between the solvents. C and H atoms of benzene molecules are shown as black and white balls, respectively. Bottom: Derived bonded-phase (gray shadow), W, MeOH, and benzene density profiles. Dashed vertical lines separate the bonded-phase region (I), the interfacial region (II), and the bulk liquid region of the pore (III).

In the first and second parts of our research program,^18,19^ we identified the key differences between W–MeOH and W–ACN mobile phases regarding the results of column equilibration and sample solvation. The solvent composition in the stationary-phase compartment as well as the immediate analyte environment in the mobile-phase compartment hold an OS density excess compared with the composition of the bulk liquid mobile phase. The amount of OS density excess is limited by the comparative strength of the W–OS hydrogen bonds,^39^ which is why W–ACN mobile phases allow for a larger OS density excess than W–MeOH mobile phases at a given OS volume fraction between 10 and 90 vol % OS.

Figure 2 illustrates the influence of the OS type on the immediate analyte environment in the mobile-phase compartment for benzene. The larger OS density excess observed with ACN as OS translates to a more favorable analyte environment, which partially explains why ACN has higher OS eluent strength than MeOH. Figure 2 also shows that, while OS density excess dominates the immediate benzene environment, W density excess is clustered above the ring plane. The aromatic πelectrons, which form weak hydrogen bonds with W and MeOH molecules,^40,41^ are hydrophilic elements in an overall apolar compound. Due to the accumulation of OS and W density excess at the hydrophobic and hydrophilic elements of a compound, respectively, the immediate analyte environment in the mobile-phase compartment is solute-specific.

**Figure 2:**
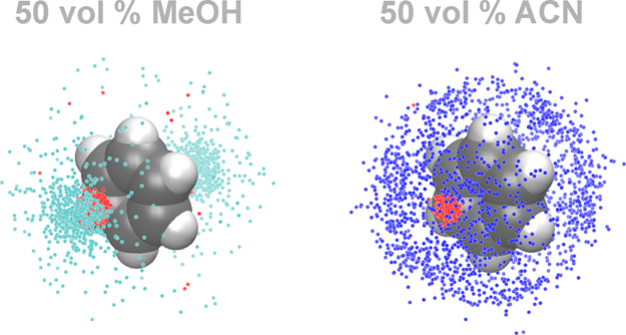
Immediate solvation environment of benzene molecules in bulk liquid W–MeOH and W–ACN mixtures containing 50 vol % OS (∼30 and 25 mol % MeOH and ACN, respectively). C and H atoms of benzene are shown as gray and white balls, respectively. Colored dots indicate local excess of W over OS density (red), MeOH over W density (cyan), and ACN over W density (blue) above the threshold value (three times the bulk liquid density of the excess solvent) as determined from the difference spatial distribution functions. The OS density excess around the benzene molecule is clearly enhanced in 50 vol % ACN compared with 50 vol % MeOH.

Adapted with permission from ref 19. Copyright 2023 American Chemical Society.

The insight that the immediate analyte environment in the mobile-phase compartment is shaped by the mobile-phase parameters as well as by solute properties, combined with the knowledge that the solvent composition in the stationary-phase compartment is a function of the mobile-phase parameters, poses the question how the mobile-phase parameters and solute properties influence the analyte environment in the stationary-phase compartment.

In the third, final part of the research program, we investigate the mobile-phase contribution to the sequential elution of sample compounds from the column through differential analyte retention by the solvated stationary phase. Our approach is to connect the experimentally observed retention behavior to changes in the composition and population of the immediate analyte environments in the solvated stationary phase as obtained from MD simulations in our RPLC slit-pore model. As the consideration of mobile-phase parameters and solute properties necessitates the analysis of large data sets, the study is divided into two parts. First, we explore the changes behind the decrease of the retention factor with increasing OS volume fraction or OS eluent strength in the mobile phase, using benzene as the simplest molecule of the ensemble to trace the different environments available to small, neutral compounds in the solvated stationary phase. Benzene has no functional groups or side chains that are known to cause different orientation preferences for an analyte molecule depending on its location in the RPLC slit pore.^22^ In the second part,^42^ we employ the full analyte ensemble to consider the dependence of the retention factor from the solute polarity (log*K*_OW_) and investigate the solute-specific response to changes in the mobile-phase parameters in view of selectivity.

## 2 Methods

### 2.1 Simulations

To generate the data for this study, we performed simulations in our RPLC slit-pore model to extend the productive trajectories obtained for benzene during the first part of the research project.^18^ Productive trajectories of 470–870 ns available for W–MeOH mobile phases from this earlier project were extended to 569–1169 ns. Productive trajectories of 433–970 ns available for W–ACN mobile phases were extended to 660–1114 ns.

The simulations for this work required a total of 100000 core hours and were performed on 144 cores of the high-performance computer HoreKa of the Steinbuch Center for Computing at the Karlsruhe Institute of Technology (Karlsruhe, Germany).

#### 2.1.1 RPLC Slit-Pore Model

The slit-pore model was constructed around a rectangular silica slab with dimensions of 12.14 nm (*x*) × 13.20 nm (*y*) × 0.93 nm (*z*), cut from β-cristobalite SiO_2_ parallel to the (111) surface, and prepared for chemical surface modification following an approach of Coasne et al.^43^ On each side of the silica slab, 300 C_18_ chains and 90 trimethylsilane groups were randomly grafted, yielding a ligand density of 3.11 μmol m^–2^ and an endcapping density of 0.93 μmol m^–2^, respectively. This procedure left 330 OH groups on each side of the silica surface, corresponding to a residual OH density of 3.42 μmol m^–2^. The silica slab was placed between 5 nm wide solvent reservoirs at the center of the simulation box. Due to the applied periodic boundary conditions, the system equals a 10 nm wide slit pore.

#### 2.1.2 Force-Field Selection

The selection of the appropriate force fields for the components of the simulation systems evolved from our previous MD simulation studies.^16–19,22,25,27–29,33,34^ Solute molecules were treated with the CHARMM general force field,^44^ W molecules were modeled with the TIP4P/2005 force field^45^ when part of W–MeOH mobile phases and with the extended simple point charge (SPC/E) force field^46^ when part of W–ACN mobile phases. The respective united-atom versions of the transferable potentials for phase equilibria (UA-TraPPE) were used for bonded-phase groups,^47^ MeOH molecules,^48^ and ACN molecules,^49^ and the silica surface was modeled with the parameters of Gulmen and Thompson.^50^

We arrived at the force-field combinations for the mobile-phase solvents through preparative bulk box simulations of W–MeOH and W–ACN mixtures conducted over the full range of OS volume fractions. Different W force fields recommended by others for W–OS mixtures^51,52^ were evaluated in combination with the respective UA-TraPPE force field for a given OS type based on how well the force-field combinations reproduced experimental data for the relevant solvent properties, that is, solvent densities and self-diffusivities, and their dependence from the OS volume fraction.^18^ The self-diffusivities turned out to be rather sensitive to the W force field, so that it was necessary to use different W force fields for W–MeOH and W–ACN mobile phases. The force-field combination for the complete simulation system has been validated by comparison with experimental OS surface excess adsorption isotherms and retention data.^18,25,33^

#### 2.1.3 Simulation Protocol

Simulations in the RPLC slit-pore model were carried out in the *NVT* ensemble (constant number of molecules *N*, simulation box volume *V*, and temperature *T*) with GROMACS version 2019.6.^53,54^ The simulation box contained ten benzene molecules and the necessary number of W and OS molecules for the equilibration of the solid–liquid interface at a given OS volume fraction in the W–MeOH or W–ACN mobile phase, determined in preparatory simulation runs (Table S1 of the Supporting Information). The achieved solute concentration of ∼0.01 mol L^–1^ reflects conditions in an analytical HPLC column.

The temperature *T* = 300 K was controlled by a Nosé–Hoover thermostat. Energy minimization was conducted with the steepest-descent method. Initial velocities were randomly assigned according to a Maxwell–Boltzmann distribution. Long-range electrostatic interactions were provided by the particle-mesh Ewald algorithm. Nonbonded interactions were modeled with a 12–6 Lennard-Jones potential. Lennard-Jones parameters for unlike interactions were obtained from the Lorentz–Berthelot combination rules. A validated cutoff radius of 1.4 nm was used for all interactions.^34^ Constraints on bonds were imposed using the GROMACS default algorithm LINCS (LINear Constraint Solver) and, for W molecules, the GROMACS implementation of the SETTLE algorithm.^55^ Productive simulations were preceded by an equilibration period of 42–82 ns. Equations of motion were integrated with a 1 fs time step. The output frequency was set to 0.5 ps.

GROMACS files for the simulation of benzene in the RPLC slit-pore model with mobile phases containing 20 vol % MeOH or 20 vol % ACN were uploaded to the Zenodo repository, along with the configuration for the empty (solvent-free) RPLC slit pore. With this information and the data given in Table S1, every simulation system of the study can be replicated.

### 2.2 Data Analysis

#### 2.2.1 Calculation of Bonded-Phase, Solvent, and Benzene Density Profiles

Density profiles were based on the number densities of the CH_2_ and CH_3_ united-atom groups of the bonded phase, the O atom of W, the O atom of MeOH, the N atom of ACN, and the center-of-mass (com) of benzene molecules, counted as a function of the distance *z* to the Si surface atoms (*z* = 0 nm). Bonded-phase density profiles, which were determined for individual alkyl groups in the C_18_ chains as well as the sum of all alkyl groups in the C_18_ chains, were calculated from 40 ns trajectories using a bin width of 0.02 nm. Solvent density profiles were calculated from 40 ns trajectories using bin widths of 0.05 and 0.1 nm for *z* ≤ 1 nm and *z* > 1 nm, respectively. Benzene density profiles were calculated from the full trajectory using a bin width of 0.05 nm.

Average solvent densities in different regions of the pore, namely, the surface-adsorbed solvent layer (*z* = 0–0.40 nm), the solvent-depleted bonded-phase region (*z* = 0.40–1.05 nm), the interfacial region (*z* = 1.05–2.55 nm), and the bulk liquid region (*z* = 2.55–5.0 nm), were calculated within the given *z*-intervals from 80 ns trajectories using a bin width of 0.05 nm.

#### 2.2.2 Determination of Chain Conformations and Conformational Parameters

C_18_ chains were categorized as (i) tilted or upright, depending on whether the distance of the first methylene group in the chain from the silica surface was *z*(CH_2_-1) ≤ 0.39 nm or *z*(CH_2_-1) > 0.39 nm, respectively, and as (ii) backfolded or extended, depending on whether the distance of the terminal methyl group in the chain from the silica surface was *z*(CH_3_-18) ≤ 0.93 nm or *z*(CH_3_-18) > 0.93 nm, respectively.

The average tilt angle of tilted and upright chains was calculated based on the bimodal distribution of the probability distribution of the angle between the surface normal and the vector pointing from the chain grafting point on the silica surface toward the first methylene group in the chain using a bin width of 1°. The average number of *gauche* defects per chain, *N*_*gauche*_, was determined for each conformational category from 40 ns trajectories. A *gauche* defect was counted when the dihedral angle between four adjacent alkyl groups in the chain (e.g., CH_2_-1 to CH_2_-4, CH_2_-2 to CH_2_-5, etc.) was <120°.

#### 2.2.3 Calculation of Benzene Distribution Coefficients and Comparison to Experimental Retention Factors

For validation of the simulated retention data, we calculated the distribution coefficients from the benzene density profiles according to eq 1, and compared the received values to experimental retention factors obtained previously for benzene at 300 K with W–MeOH and W–ACN mobile phases containing between 20 and 90 vol % OS on a silica-based, endcapped, C_18_ column using uracil as dead time marker.^19^

The definitions of the distribution coefficient and the retention factor in eq 1 imply that the border between the stationary phase and the mobile phase is known, so that the values of *c*_SP_, *c*_MP_, *V*_SP_, and *V*_MP_ can be determined with certainty, but that is not the case at all. The solvent-accessible volume in a column can be determined experimentally,^56^ but this includes the volume occupied by the solvent molecules in the stationary phase and is therefore larger than *V*_MP_ (by an unspecified amount). In HPLC practice, the value of *V*_MP_ is estimated with more or less accuracy, for example, through dead time markers, whereas in molecular simulations, the border between the stationary phase and the mobile phase needs to be defined.^57^

We previously introduced a definition for the stationary-phase border based on contacts between analyte molecules and bonded-phase groups, as the idea of analyte retention revolves around solute interaction with the stationary phase.^25^ The location of the stationary-phase limit, *z*_SP_, was taken as the distance from the silica surface where an analyte molecule had–averaged over a 40 ns trajectory–less than one bonded-phase contact. We mention here that the threshold value for *z*_SP_ is arbitrary and may eventually have to be refined when larger analyte compounds are introduced.

The benzene distribution coefficient was then calculated as the ratio between the averaged benzene density at *z* ≤ *z*_SP_ and the averaged benzene density at *z* > *z*_SP_. The received *K* values are shown in Figure 3 along with the experimental retention factors. The simulated distribution coefficients excellently capture the experimentally observed dependence of the retention factor from the OS volume fraction and OS type in the mobile phase, which validates our definition of the stationary-phase border as well as the force-field selection.

**Figure 3:**
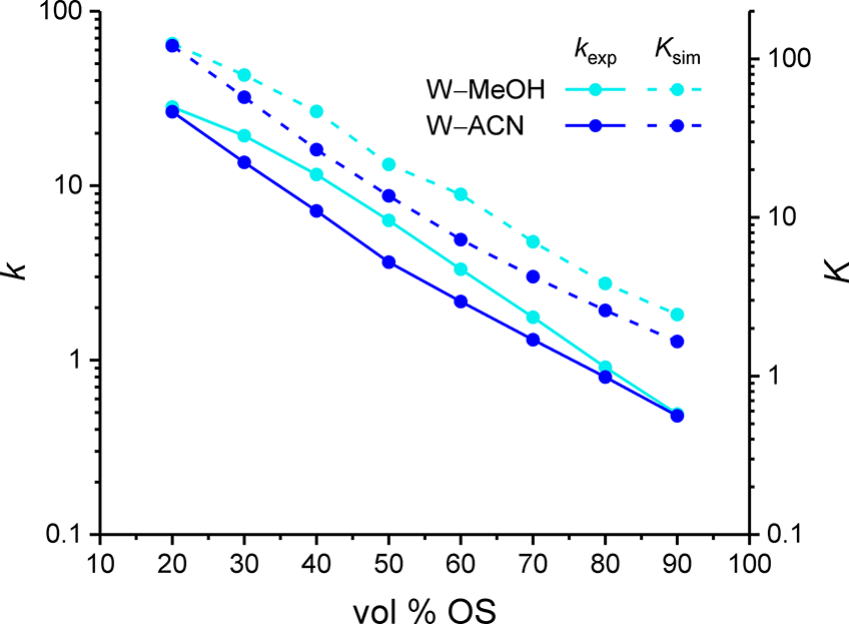
Benzene retention on a silica-based, endcapped, C_18_ stationary phase with W–MeOH or W–ACN mobile phases. Experimental retention factors *k* obtained using uracil as dead time marker (solid lines) are compared with distribution coefficients *K* derived from simulations in the RPLC slit-pore model using *z*_SP_ as the stationary-phase limit (dashed lines).

Experimental data reproduced with permission from ref 19. Copyright 2023 American Chemical Society.

#### 2.2.4 Calculation of Bonded-Phase, W, and OS Contacts for Benzene Molecules in the Solvated Stationary Phase

The calculation of benzene contacts relied on cutoff radii derived from radial distribution functions (RDFs) calculated pairwise between the com of benzene molecules and the united-atom groups of the bonded phase, the O atom of W molecules, the O atom of MeOH molecules, or the N atom of ACN molecules. RDFs calculated for different OS volume fractions in a given W–OS mobile phase were averaged, and the cutoff radius was determined as the location of the minimum following the first maximum in the averaged RDF. Benzene–bonded-phase RDFs, calculated in the slit-pore model, yielded a cutoff radius of *r*_BP_ = 0.79 nm. Cutoff radii for benzene–solvent contacts, *r*_W(O)_ = 0.63 nm, *r*_MeOH(O)_ = 0.70 nm, and *r*_ACN(N)_ = 0.67 nm, were available from the second part of the research project.^19^

Contact profiles for benzene, *C*_BP_(*z*), *C*_W_(*z*), *C*_MeOH_(*z*), and *C*_ACN_(*z*), which indicate the number of contacts with the designated species per benzene molecule at a given distance *z* from the silica surface, were constructed from 40 ns trajectories using a bin width of 0.05 nm. At each distance *z*, the number of bonded-phase groups, W, MeOH, or ACN molecules within a radius of *r ≤ r*_BP_, *r*_W(O)_, *r*_MeOH(O)_, or *r*_ACN(N)_, respectively, was counted and the received value then normalized by the benzene density in the respective bin.

The average number of contacts per benzene molecule in a section of the stationary phase (i.e., the partitioning or adsorption peak) or the entire stationary phase was calculated by summing the product of *C*_BP_(*z*) and ρ_analyte_(*z*) per bin over all bins in the *z*-interval of interest. The received value was then divided by the sum of ρ_analyte_(*z*) in the respective *z*-interval, as determined from the whole trajectory. Section-averaged contacts are indicated by angle brackets, stationary phase-averaged contacts are indicated by angle brackets with the subscript SP.

Standard deviations for <*C*_BP_>_SP_, <*C*_W_>_SP_, and <*C*_OS_>_SP_ were between ±0.85 and ±1.77, between ±0.06 and ±0.32, and between ±0.11 and ±0.40, respectively, with W–MeOH mobile phases, and between ±0.83 and ±1.83, between ±0.07 and ±0.21, and between ±0.12 and ±0.35, respectively, with W–ACN mobile phases.

## 3 Results and Discussion

### 3.1 Solvation of the Stationary Phase

Figure 1 conveys that the immediate environment of a small analyte molecule in the solvated stationary phase depends on the local density of bonded-phase groups and solvent molecules and thus on how the bonded-phase and solvent density is distributed over the stationary-phase extension. The bonded-phase density distribution is informed by the conformation of the C_18_ chains, which is, in turn, sensitive to the mobile-phase parameters, as we will show below. We begin the presentation of our results by reviewing the consequences of increasing mobile-phase elution strength for the stationary-phase solvation. The structure of the solvent density profiles was extensively discussed in connection with the OS surface excess adsorption isotherms in the first part of our research program,^18^ so we keep explanations brief. Here, we identify the solvent density changes that occur at characteristic locations in the system upon an increase of the OS volume fraction or eluent strength in the mobile phase.

A glance at the W and OS density profiles, reproduced in Figure 4, shows that raising the OS volume fraction in the mobile phase decreases the W density and increases the OS density everywhere in the system, which comprises the bulk liquid region (*z* > 2.55 nm), the interfacial region (*z* = 1.05–2.55 nm), and the bonded-phase region (*z* ≤ 1.05 nm) with the surface-adsorbed solvent layer (*z* ≤ 0.4 nm). The consequences of increasing OS eluent strength are less easily anticipated, not least because the two mobile-phase compositions are compared at equal OS volume fraction, as common in HPLC practice, but also because the consequences are specific to a region. Figure 5 therefore directly compares the average W and OS densities that are obtained in the above-cited regions with W–MeOH vs W–ACN mobile phases.

**Figure 4:**
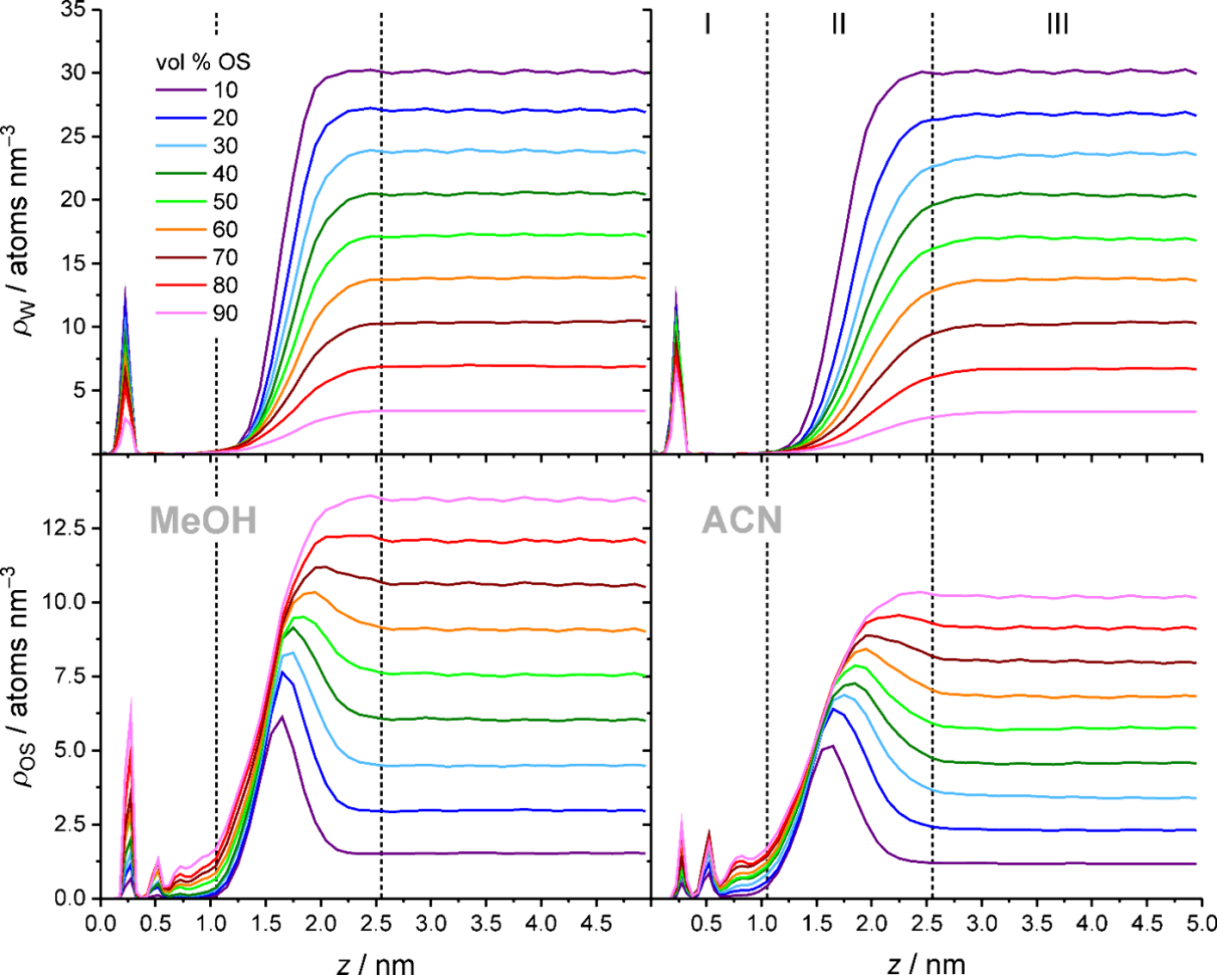
Evolution of W (top) and OS density profiles (bottom) in the RPLC slit-pore model with increasing OS volume fraction in the W–MeOH (left) or W–ACN mobile phase (right). Dashed vertical lines separate the bonded-phase region (I), the interfacial region (II), and the bulk liquid region of the pore (III).

Adapted with permission from ref 18. Copyright 2022 American Chemical Society.

**Figure 5:**
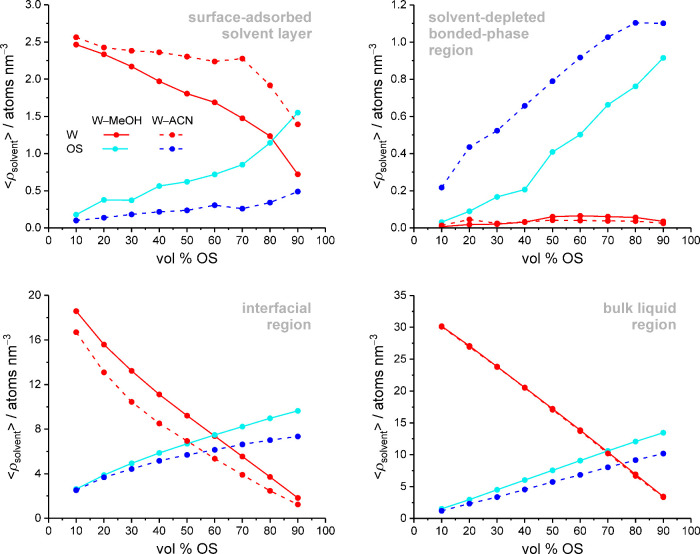
Evolution of the average W and OS densities in different regions of the pore with increasing OS volume fraction in the W–MeOH or W–ACN mobile phase.

Because MeOH has a smaller molecular volume than ACN, the OS (number) density in the bulk liquid region increases with the OS eluent strength while the W density remains the same. As explained in the Introduction, the solvent composition in the interfacial region depends on the amount of W molecules that are dragged by OS molecules through W–OS hydrogen bonds into the bonded-phase chains. The W density in the interfacial region is therefore a function of the OS density in the interfacial region, which is higher for MeOH due to its smaller molecular volume, and of the number of W–OS hydrogen bonds per OS molecule, which is also higher for MeOH than ACN. The W and OS density in the interfacial region thus both decrease with increasing OS eluent strength. The higher W density observed with W–MeOH mobile phases in the interfacial region, which holds >90% of solvent molecules in the solvated stationary phase,^18^ explains why the system contains more W molecules with a W–MeOH than a W–ACN mobile phase of equal OS volume fraction. (This is reflected by the number of W and OS molecules in the simulation box, Table S1, and by the average W and OS densities in the solvated stationary phase, Table S2.)

In the bonded-phase region, W density is essentially confined to the surface-adsorbed solvent layer. These surface-bound W molecules have considerably longer residence times than W molecules in the interfacial region, as previously shown.^58^ Because ACN molecules contribute less to the coordination of residual OH groups than MeOH molecules, the W density in the surface-adsorbed solvent layer increases while the OS density decreases with increasing OS eluent strength. The next density peak in the bonded-phase region, at *z* = 0.5 nm, consists of solvent molecules that form hydrogen bonds with W molecules of the surface-adsorbed solvent layer. Higher W density in the surface-adsorbed solvent layer entails higher OS density in the adjacent, second solvent layer. The less defined solvent density peaks in the bonded-phase region (*z* = 0.6–1.05 nm) represent OS molecules that solvate bonded-phase groups. ACN has better solvation properties than MeOH for alkyl groups, as, for example, reflected by the analyte solvation shells in W–MeOH and W–ACN mixtures (cf. Figure 2).^19^ The solvent-depleted part of the bonded-phase region (*z* = 0.4–1.05 nm) is therefore the only location in the system, where the OS density is larger with W–ACN than W–MeOH mobile phases.

According to Figure 5, which serves as a graphic summary of the mobile phase-induced changes in the stationary-phase solvation, increasing the mobile-phase elution strength, whether via the OS volume fraction or the OS eluent strength, generally entails higher OS density in the solvent-depleted part of the bonded-phase region and lower W density in the interfacial region.

### 3.2 Conformation of the Bonded Phase

For the most extended and homogeneous distribution of bonded-phase density in the stationary-phase compartment, the C_18_ chains need to be upright and free of *gauche* defects, but in reality the chains deviate more or less from this ideal, depending on the mobile-phase parameters. Relevant aspects of the mobile-phase sensitivity of the bonded-phase conformation were already established by Siepmann and co-workers, who conducted a series of Monte Carlo simulations in a slit-pore model of a silica-based, C_18_ stationary phase equilibrated with W–MeOH and W–ACN mobile phases at selected OS mole fractions as well as with the neat solvents.^15,59^ From these studies, we know that the average distance of the chain end groups from the silica surface, *z*(CH_3_-18), increases with the OS mole fraction and the OS eluent strength in the mobile phase and from neat W to neat MeOH to neat ACN. Taken together, these observations already indicate that the bonded-phase density extends toward the bulk liquid region with increasing mobile-phase elution strength. Our analysis focuses on the details of the bonded-phase response to the solvation of the stationary phase and the consequences for the analyte density distribution.

Figure 6, which shows the density distribution of the chain end groups, confirms that the bonded phase extends with increasing mobile-phase elution strength. Moreover, the chain end density profiles reveal two major conformational categories, which we denote as backfolded or extended chains, depending on whether the chain end group is predominantly located in the bonded-phase region or the interfacial region, respectively. Importantly, Figure 6 visualizes that an increase of the mobile-phase elution strength, whether from an increase of the OS volume fraction or the OS eluent strength, entails a redistribution of bonded-phase density toward larger *z*-values.

**Figure 6:**
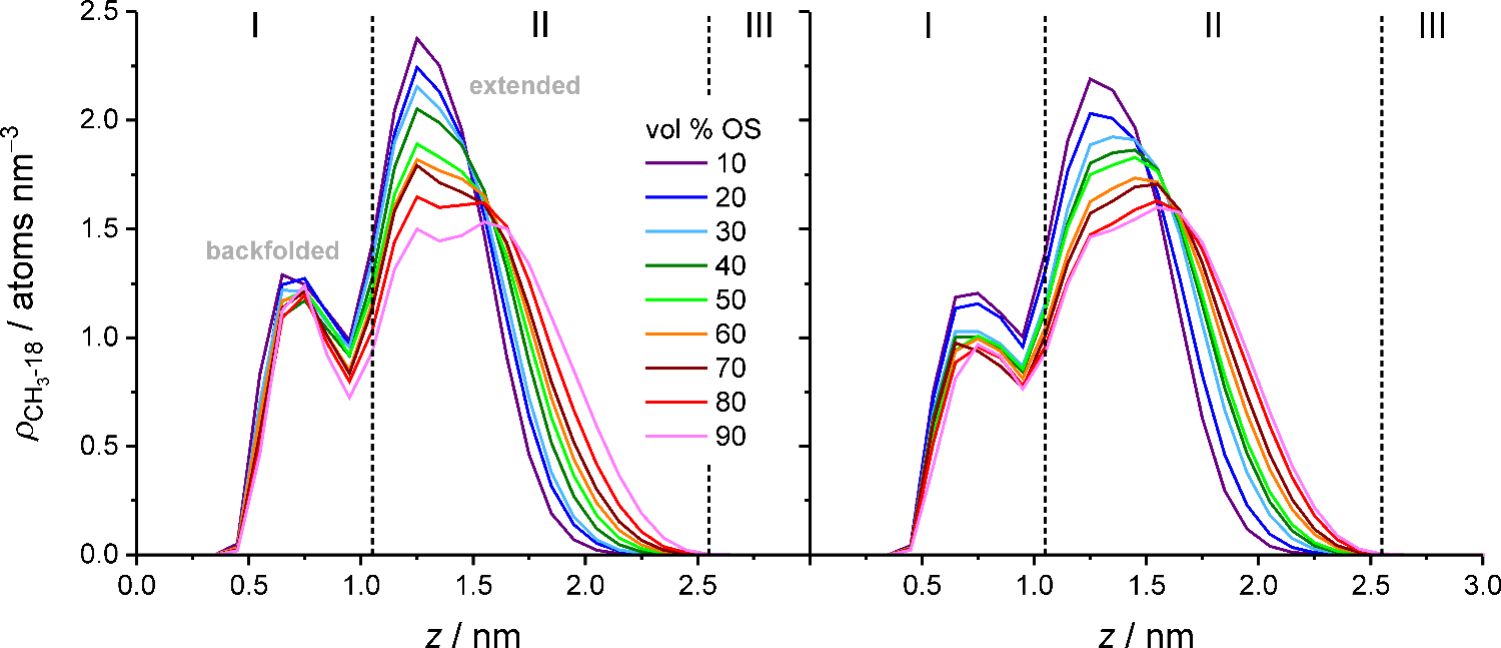
Evolution of C_18_ chain end density profiles with increasing OS volume fraction in the W–MeOH (left) or W–ACN mobile phase (right). Dashed vertical lines separate the bonded-phase region (I), the interfacial region (II), and the bulk liquid region of the pore (III).

Figure 7 shows that the bimodal distribution of the chain end density is mirrored by the bimodal distribution of the chain tilt angle, formed between the grafting vector (the vector pointing from the chain grafting point on the silica surface to the first methylene group in the chain) and the surface normal.^60^ The C_18_ chains either tilt away from the surface normal (average tilt angle of 32.9°) or nearly align with the surface normal (average tilt angle of 9.0°) and are thus upright. Backfolded and extended chains can be tilted or upright, yielding four conformational categories.

**Figure 7:**
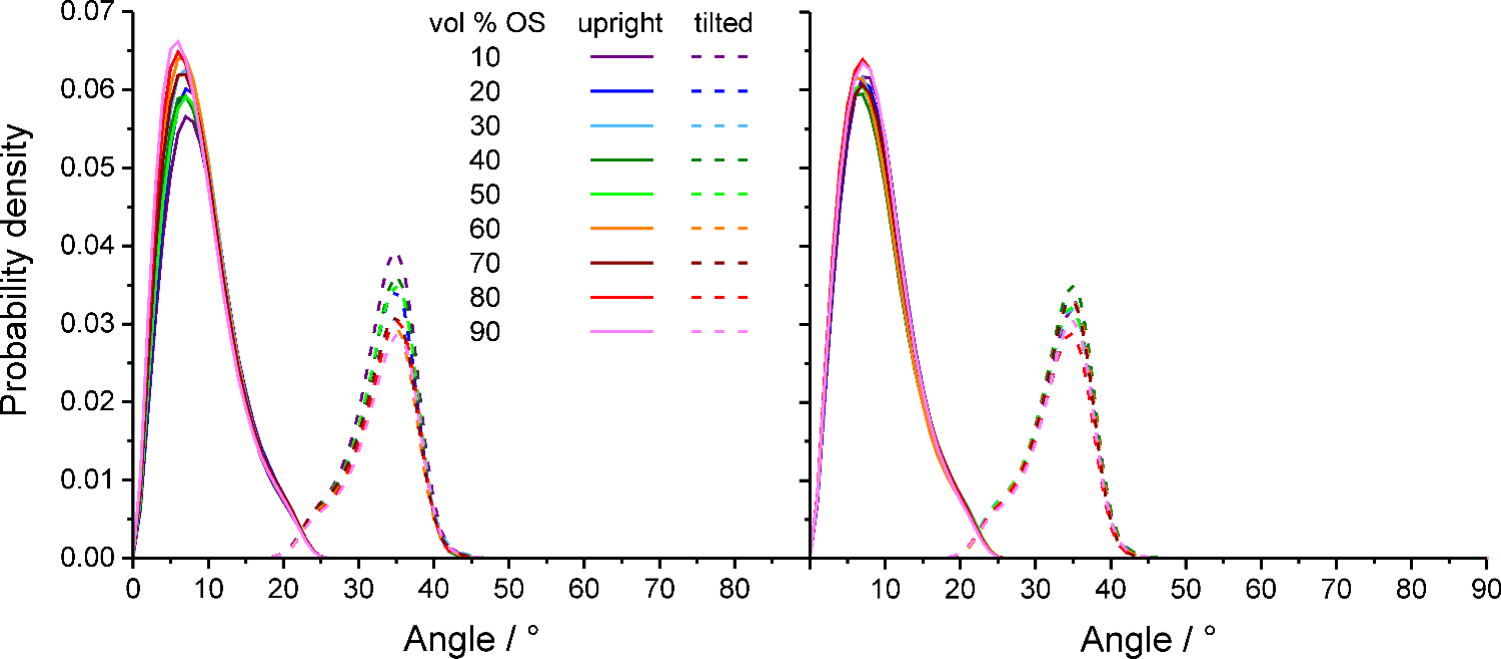
Evolution of the probability distribution for the chain tilt angle with increasing OS volume fraction in the W–MeOH (left) or W–ACN mobile phase (right).

Table 1 details the contributions from each category to the overall bonded-phase conformation and summarizes the evolution of the conformational parameters, namely *z*(CH_3_-18) and the number of *gauche* defects per chain, *N*_*gauche*_, between 10 and 90 vol % OS in the mobile phase. (Tables S3–S6 list the contributions from each category and the associated conformational parameters for every OS volume fraction.) Upright chains and extended chains contribute more to the bonded-phase conformation than tilted chains and backfolded chains, which makes upright, extended chains the dominant conformational category. The data in Table 1 show that the overall bonded-phase extension, as judged by the value of *z*(CH_3_-18)_all_, profits from a high fraction of upright, extended chains, which, in turn, increases with the mobile-phase elution strength.

**Table 1:** Bonded-Phase Response to Increasing Mobile-Phase Elution Strength

bonded-phase property		W–MeOH		W–ACN	
		10 vol % OS	90 vol % OS	10 vol % OS	90 vol % OS
*z*(CH_3_-18)_all_		1.18 nm	1.34 nm	1.20 nm	1.39 nm
*N*_*gauche*,all_		3.94	3.92	3.96	3.81
				
*f*_bf_ ^a^		26.2%	21.6%	25.0%	18.3%
*f*_ext_ ^b^		73.8%	78.4%	75.0%	81.7%
*f*_tilted_ ^c^		35.2%	26.6%	28.5%	27.4%
*f*_upright_ ^d^		64.8%	73.4%	71.5%	72.6%
*f*_bf,tilted_ ^a^ ^,^ ^c^		9.6%	5.8%	6.8%	5.2%
	*z*(CH_3_-18)	0.72 nm	0.73 nm	0.73 nm	0.75 nm
	*N*_*gauche*_	3.92	3.92	3.96	3.86
				
*f*_bf,upright_ ^a^ *^,^ ^d^*		16.7%	15.9%	18.2%	13.1%
	*z*(CH_3_-18)	0.73 nm	0.74 nm	0.73 nm	0.74 nm
	*N*_*gauche*_	4.18	4.25	4.19	4.17
				
*f*_ext,tilted_ ^b^ ^,^ ^c^		25.6%	20.8%	21.7%	22.2%
	*z*(CH_3_-18)	1.34 nm	1.51 nm	1.36 nm	1.52 nm
	*N*_*gauche*_	3.73	3.62	3.74	3.58
				
*f*_ext,upright_ ^b^ ^,^ ^d^		48.2%	57.5%	53.3%	59.5%
	*z*(CH_3_-18)	1.34 nm	1.53 nm	1.36 nm	1.55 nm
	*N*_*gauche*_	3.98	3.94	3.97	3.82

a *z*(CH_3_-18) ≤ 0.93 nm.

b *z*(CH_3_-18) > 0.93 nm.

c *z*(CH_2_-1) ≤ 0.39 nm.

d *z*(CH_2_-1) > 0.39 nm.

The top two rows in Table 1 show that the increase of *z*(CH_3_-18)_all_ with increasing mobile-phase elution strength is not necessarily reflected by a concomitant decrease of *N*_*gauche*,all_. Contrary to expectation, the value of *N*_*gauche*,all_ is not a sensitive indicator for the mobile-phase influence on the bonded-phase conformation, as already observed previously.^15^ A survey of the data compiled in Table 1 reveals that the bonded-phase response to the mobile-phase elution strength is, in detail, quite complex. The value of *z*(CH_3_-18)_all_ is limited by the fraction of backfolded chains, which are much shorter than extended chains and whose conformation is largely insensitive to the mobile-phase elution strength. The value of *N*_*gauche*,all_ is primarily limited by the fraction of upright chains, which have a higher number of *gauche* defects than tilted chains.

At 10 vol % OS, the values for *z*(CH_3_-18)_all_ and *N*_*gauche*,all_ are slightly lower with the W–MeOH mobile phase, which generates a larger fraction of backfolded chains as well as a larger fraction of tilted chains than the W–ACN mobile phase. The value for *z*(CH_3_-18)_all_ increases with increasing OS volume fraction in the mobile phase, as (i) backfolded chains are partially converted into extended chains, and (ii) extended chains are stretched through an improvement of the dihedral angles. Independent of the OS volume fraction, extended chains tend to have larger *z*(CH_3_-18) and lower *N*_*gauche*_ values with W–ACN mobile phases. Additionally, W–MeOH mobile phases favor the conversion of tilted into upright chains with increasing OS volume fraction, whereas W–ACN mobile phases favor the conversion of backfolded into extended chains. At 90 vol % OS, the difference between W–MeOH and W–ACN mobile phases in the fraction of backfolded chains has grown and the difference in the fraction of tilted chains has leveled out, so that the value for *z*(CH_3_-18)_all_ is now clearly larger, and the value for *N*_*gauche* *,*all_ clearly lower, with the W–ACN mobile phase.

Except for the conversion of tilted into upright chains, which concerns the CH_2_-1 groups that are located in a narrow interval near the silica surface (*z* = 0.25–0.5 nm), the observed conformational changes cannot be assigned with certainty to local solvent density changes. First, the individual alkyl groups of the C_18_ chains are distributed over wide *z*-intervals, which makes the assignment of a dihedral angle to a specific location difficult. Second, the solvation-induced improvement of a dihedral angle may move along the chain and affect dihedral angles further up the chain. It is also conceivable that local solvent density changes trigger dihedral angle changes at another location. The comprehensive analysis of the conformational data indicates that the increase of OS density in the solvent-depleted bonded-phase region and the decrease of W density in the interfacial region favor the various improvements of the chain conformation. This effect is probably assisted by the properties of the OS type, particularly (but not necessarily exclusively), the molecular volume of the OS.

In summary, the local solvent density changes in the solvated stationary phase associated with increasing mobile-phase elution strength favor the conversion of backfolded into extended chains, which shifts bonded-phase density from the bonded-phase region to the interfacial region, and stretch out extended chains, which redistributes and extends the bonded-phase density in the interfacial region toward the bulk liquid region.

### 3.3 Analyte Density Distribution in the Solvated Stationary Phase

In the chromatographic literature, analyte density in the bonded-phase region has traditionally been associated with solute partitioning into the bonded phase, whereas analyte density in the interfacial region has been associated with solute adsorption to the bonded phase.^13,21^ Solute partitioning describes the transition from the predominantly aqueous bulk liquid region (up to ∼70 vol % OS, the mobile phase contains more W than OS molecules) to a solvent-depleted, hydrophobic environment, whereas solute adsorption describes the transition to a predominantly hydrophobic environment that allows for solute contact with the bulk liquid. Solute partitioning into the bonded-phase region differs, however, from solute partitioning between two immiscible liquids, as molecular simulations have confirmed that the diffusive mobility in the bonded-phase region is low. In contrast, solute adsorption to the bonded phase is typically accompanied by a mobility gain, as the OS density excess in the interfacial region favors the diffusive mobility.^18,22,34–37^

The benzene density profiles in Figure 8 show that the adsorption peak holds more analyte density than the partitioning peak, which reflects a preference for solute adsorption over solute partitioning. Importantly, the same preference has so far been observed for every small compound included in molecular simulation studies (i.e., for solutes that allow the observation of two separate density peaks).^21–28,30–32,57,60,61^ The preference for solute adsorption over solute partitioning has been attributed to the lower entropic cost of cavity formation in the interfacial region compared with the bonded-phase region.^13^ In any case, the preference of small solute molecules for a specific region is not a question of spatial restrictions. Although narrower than the interfacial region, the bonded-phase region is also free from spatial restrictions for small solutes. The simulation of the ethylbenzene adsorption isotherm in the RPLC slit-pore model with a mobile phase containing 60 vol % ACN revealed that the partitioning contribution to the ethylbenzene density in the stationary phase loses only 2% (within the statistical error) over an analyte concentration range from 0.01 to 0.14 mol L^–1^.^62^ The 2% loss is incompatible with the presence of spatial restrictions in the bonded-phase region at conditions that represent the linear part of the analyte adsorption isotherm.

**Figure 8:**
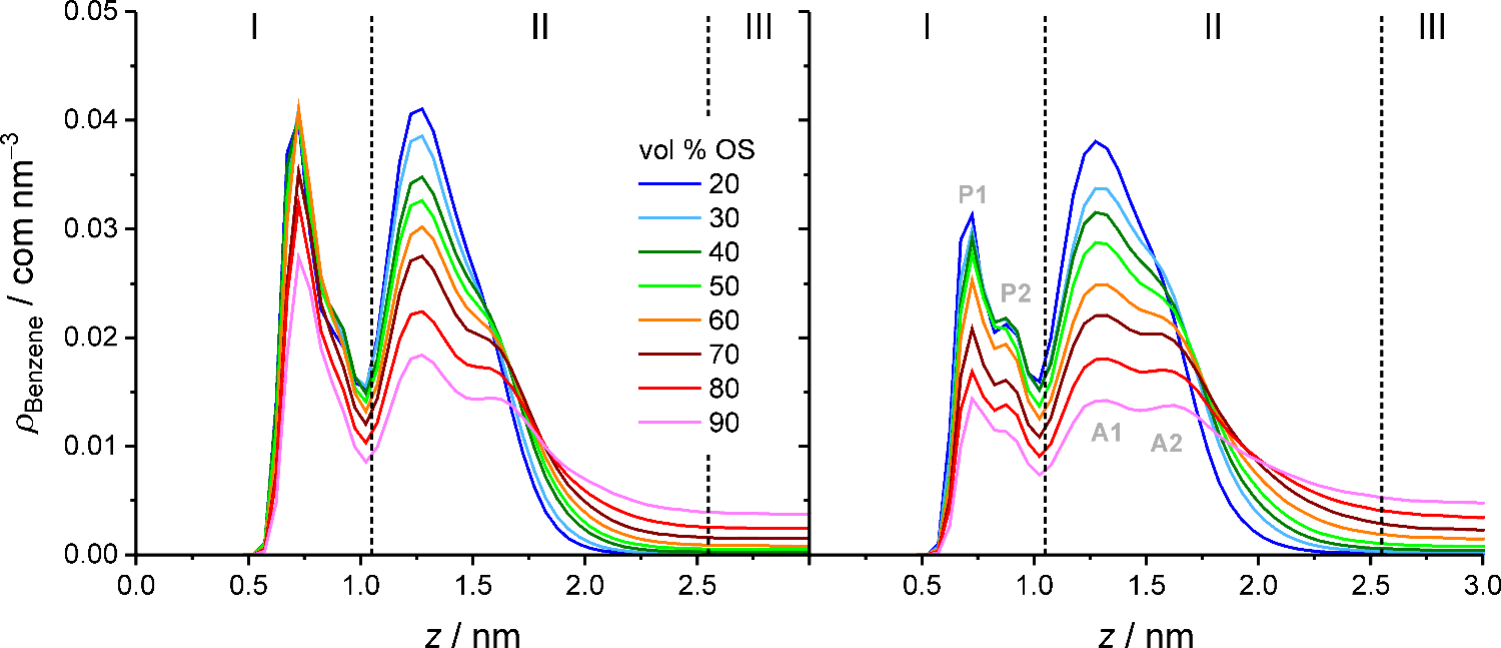
Evolution of the benzene density distribution in the solvated stationary phase with increasing OS volume fraction in the W–MeOH (left) or W–ACN mobile phase (right). Dashed vertical lines separate the bonded-phase region (I), the interfacial region (II), and the bulk liquid region of the pore (III). P1 and P2 designate the silica-surface side and the bulk-liquid side of the partitioning peak in the bonded-phase region, respectively, and A1 and A2 designate the silica-surface side and the bulk-liquid side of the adsorption peak in the interfacial region, respectively.

The comparison of Figures 8 and 6 shows that the analyte density mirrors the chain end density in responding to increasing mobile-phase elution strength. (Please note that, due to the size difference between the solute molecule and the methyl group, the benzene density profiles are shifted to slightly larger *z*-values compared with the chain end density profiles.) This means that the shift of analyte density from the solvated stationary phase to the bulk liquid region (and thus a lower distribution coefficient and retention factor) is accompanied by an analyte density shift within the solvated stationary phase.

The redistribution and extension of benzene density toward larger *z*-values is reflected by the development of a second maximum in the adsorption peak, a density shift toward the second maximum within the adsorption peak, and the extension of the adsorption peak toward the bulk liquid region. Additionally, benzene density may be shifted from the partitioning to the adsorption peak, and thus from the bonded-phase to the interfacial region. This occurs when the OS eluent strength is increased, as recognizable from the visual comparison of the benzene density profiles obtained with W–MeOH vs W–ACN mobile phases in Figure 8, but not necessarily when the OS volume fraction is increased. Averaged over the investigated vol % OS range, the partitioning peak contributes 34% and 27% of the benzene density in the stationary phase with W–MeOH and W–ACN mobile phases, respectively. A similar-sized density shift is observed with W–ACN mobile phases, where the partitioning contribution declines steadily from 30% to 22% between 20 and 90 vol % ACN. With W–MeOH mobile phases, however, the partitioning contribution drops only at >60 vol % MeOH.

In summary, increasing the mobile-phase elution strength entails a redistribution of analyte density within the solvated stationary phase, whereby the extent and details of this process depend on the OS type in the mobile phase. Analyte density in the interfacial region is shifted and extended toward the bulk liquid region with increasing OS volume fraction in the mobile phase; W–ACN mobile phases engender an additional redistribution of benzene density from the bonded-phase to the interfacial region.

### 3.4 Local Analyte Environments in the Solvated Stationary Phase

To elucidate the composition of the different analyte environments behind the benzene density profiles in Figure 8, we defined the extension of an immediate analyte environment analogously to the first coordination shell of a solute molecule in bulk liquids.^19^ First, we derived sphere radii for each contacting species (i.e., for bonded-phase groups and for W, MeOH, and ACN molecules) from the respective RDFs calculated pairwise between benzene molecules and the contacting species. The radii *r*_BP_, *r*_W_, *r*_MeOH_, and *r*_ACN_ define the first coordination shell of benzene molecules with respect to the contacting species. Because the radii depend on the size of the contacting species as well as on the specific atom that was used to represent the contacting species in the RDF, an immediate analyte environment does not refer to one sphere volume, but to three sphere volumes.

To define the immediate benzene environment at a given location in the solvated stationary phase, the number of contacting species within the respective sphere volume was counted (i.e., the number of bonded-phase groups within *r* ≤ *r*_BP_, the number of W molecules within *r* ≤ *r*_W_, etc.) and the resulting values were then normalized by the benzene density at this location. *C*_BP_(*z*), *C*_W_(*z*), and *C*_OS_(*z*) give the number of contacts per benzene molecule at a given distance *z* from the silica surface with bonded-phase groups, W molecules, and OS molecules, respectively.

Figure 9 provides the spatially resolved contact data for benzene molecules in the solvated stationary phase. For visual simplicity, W and OS contacts were combined. The solvent contacts follow a straightforward increase from the bonded-phase region through the interfacial region until they recover constant values in the bulk liquid region of the pore. The bonded-phase contacts start with high values in the bonded-phase region and then decrease smoothly over the extension of the interfacial region to zero at *z* > *z*_SP_. Figure 9 shows what could have been gleaned from the bonded-phase and solvent density profiles of Figure 1, namely that the immediate analyte environment in the solvated stationary phase, which is dominated by bonded-phase groups up to *z* ≈ 1.9 nm or ∼75% of the distance between silica surface and bulk liquid region, loses bonded-phase groups and gains solvent molecules with increasing distance from the silica surface. As a consequence, partitioned analyte molecules have decidedly more bonded-phase and fewer solvent contacts than adsorbed analyte molecules.

**Figure 9:**
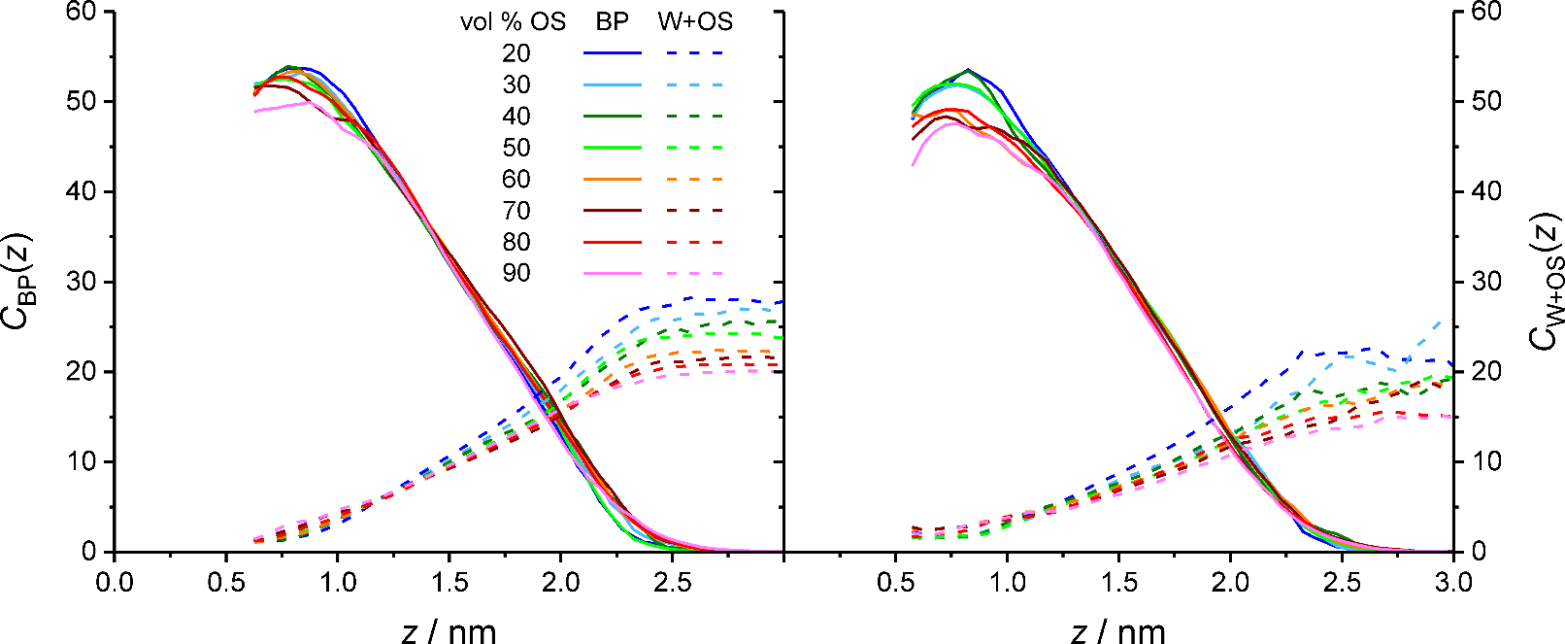
Evolution of the spatial distribution of bonded-phase and solvent contacts per benzene molecule in the solvated stationary phase with increasing OS volume fraction in the W–MeOH (left) or W–ACN mobile phase (right).

To define the composition of the immediate analyte environments associated with solute partitioning and solute adsorption, we divided the solvated stationary phase into four sections around the benzene density maxima (cf. Figure 8) and calculated the average number of benzene contacts with bonded-phase groups, W molecules, and OS molecules within each section as <*C*_BP_>, <*C*_W_>, and <*C*_OS_>, respectively. The resulting section-averaged contact data in Figure 10 reflect at once the dependence of the benzene contacts from the location in the solvated stationary phase and from the OS volume fraction and OS eluent strength in the mobile phase.

**Figure 10:**
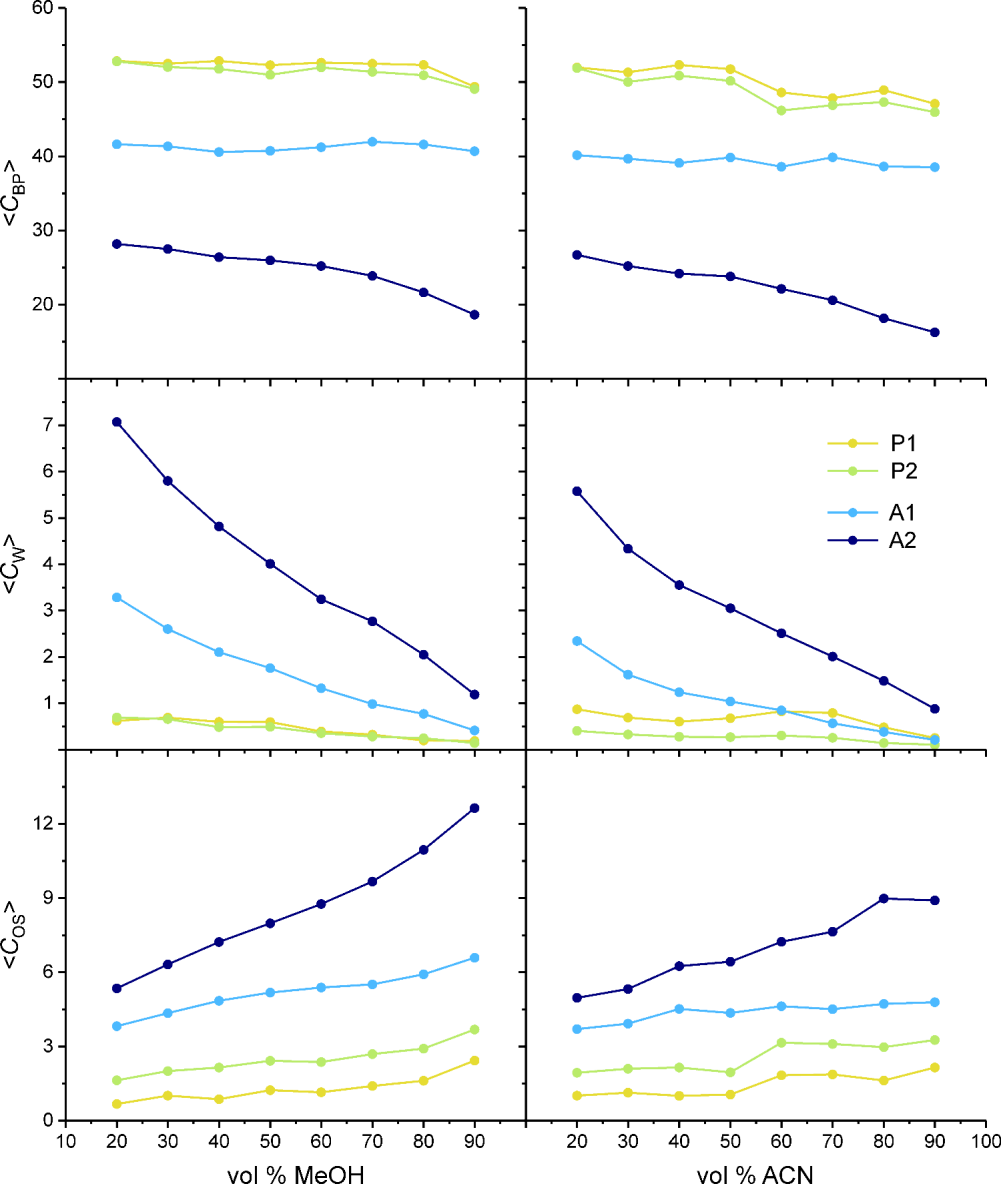
Evolution of the immediate analyte environment in different sections of the solvated stationary phase with increasing OS volume fraction in the W–MeOH (left) or W–ACN mobile phase (right). The composition of the immediate analyte environments is quantified by the section-averaged number of contacts with bonded-phase groups, W molecules, and OS molecules per benzene molecule (top, middle, and bottom, respectively). Peak abbreviations as in Figure 8.

Figure 10 proves that the composition of an analyte environment depends foremost on its location in the solvated stationary phase. The two sections in the bonded-phase region (partitioning peaks P1 and P2) represent highly similar environments that are largely insensitive to the mobile-phase parameters. The two sections in the interfacial region (adsorption peaks A1 and A2), on the other hand, represent analyte environments that differ strongly in composition and mobile-phase sensitivity. Regarding the value of <*C*_BP_> and the mobile-phase sensitivity of <*C*_BP_> and <*C*_OS_>, A1 is more similar to P1 and P2 than to A2. Actually, A1 shows the least sensitivity of <*C*_BP_> to the mobile-phase parameters among the four sections. A1 and A2 show both a strong dependence of <*C*_W_> from the mobile-phase elution strength, which reflects the associated decrease of W density in the interfacial region, but A2 is additionally highly sensitive to the mobile-phase elution strength regarding <*C*_BP_> and <*C*_OS_>. Consequently, the change in the composition of the immediate environment experienced by an analyte molecule over the extension of the interfacial region becomes more pronounced with increasing mobile-phase elution strength.

The sensitivity of <*C*_BP_> to the mobile-phase elution strength is related to the associated changes in the bonded-phase conformation (cf. Table 1). The comparatively small decrease of <*C*_BP_> observed for P1 and P2 in Figure 10 originates from the conversion of backfolded into extended chains associated with increasing OS density in this region. As pointed out above, this conversion shifts bonded-phase density from the bonded-phase to the interfacial region, where, at the same time, bonded-phase density is redistributed toward the bulk liquid region owing to the conformational improvement of extended chains. Thus, the silica-surface side of the adsorption peak maintains a fairly constant bonded-phase density at increasing mobile-phase elution strength, which is reflected by the almost constant <*C*_BP_> value observed for A1. The strongest decline of <*C*_BP_> (and concurrent rise of <*C*_W_> and <*C*_OS_>) with increasing mobile-phase elution strength occurs in A2. The width of this section increases with the mobile-phase elution strength (as opposed to the constant width of P1, P2, and A1), enabling an analyte density shift within this section to larger *z*-values with lower bonded-phase and higher solvent density.

Unsurprisingly, the <*C*_W_> and <*C*_OS_> values in Figure 10 reflect the respective W and OS densities in the solvated stationary phase (cf. Figures 4 and 5). For P1 and P2, the difference in solvent contacts between the two mobile-phase compositions is small, because solvent contacts contribute little to the immediate analyte environment in the bonded-phase region. The <*C*_W_> and <*C*_OS_> values for A1 and A2 are generally higher with W–MeOH than W–ACN mobile phases, as are the average W and OS densities in the interfacial region (Figure 5). At the same time, the <*C*_BP_> values for A1 and A2 are also higher with W–MeOH than W–ACN mobile phases. (As these details may be difficult to recognize from Figure 10, the section-averaged values for <*C*_BP_>, <*C*_W_>, and <*C*_OS_> were redrawn to enable the direct comparison of the benzene contacts observed with W–MeOH vs W–ACN mobile phases in Figures S1–S3 of the Supporting Information.)

The latter observation has important implications. If only the variation of the OS volume fraction were considered, the data in Figure 10 could be interpreted as indicating that the <*C*_BP_> values in the interfacial region decrease as the corresponding <*C*_OS_> values increase. But the data received for the variation of the OS eluent strength prove that higher <*C*_OS_> values are not incompatible with higher <*C*_BP_> values. This contradicts the notion that an influx of OS molecules into the solvated stationary phase releases analyte molecules into the bulk liquid region by replacing analyte–bonded-phase contacts with analyte–OS contacts and thus rejects the idea of competition between analyte and OS molecules for bonded-phase contacts as an explanation for the mobile-phase elution strength.

In summary, the immediate analyte environment changes gradually over the extension of the solvated stationary phase and reflects primarily the density distribution of the bonded phase and secondarily the solvation of the stationary phase. In the bonded-phase region (solute partitioning), analyte molecules have many bonded-phase and few solvent contacts. Over the extension of the interfacial region (solute adsorption), analyte molecules lose bonded-phase and gain solvent contacts. The mobile-phase sensitivity of an analyte environment increases strongly toward the bulk liquid region, which means that a variation of the mobile-phase parameters affects mainly the composition of the analyte environments in the interfacial region. Importantly, a loss of bonded-phase contacts observed with increasing mobile-phase elution strength for analyte molecules in the adsorption peak is generally accompanied by a loss of W contacts, but not necessarily by a gain of OS contacts.

### 3.5 Average Analyte Environment in the Solvated Stationary Phase

In the preceding section we relied on spatially resolved contact data to quantify how the distance-dependent heterogeneity of the solvated stationary phase shapes the local analyte environment. To regain the macroscopic perspective of HPLC practice, where the retention factor refers to the analyte density distribution between the stationary-phase and mobile-phase compartment of a column (cf. eq 1), we calculated the number of contacts with bonded-phase groups, W molecules, and OS molecules per benzene molecule averaged over the extension of the solvated stationary phase (*z* ≤ z_SP_) as a measure for the average analyte environment in the solvated stationary phase. The stationary phase-averaged contacts quantify at once the composition of the different analyte environments in the solvated stationary phase and the benzene density in these environments. The value of <*C*_BP_>_SP_ depends therefore on the relative population of analyte environments with different bonded-phase density by benzene molecules.

The redistribution of analyte density to larger *z*-values with increasing mobile-phase elution strength observed in Figure 8 indicates the progressive population of analyte environments with lower bonded-phase density and thus predicts a decrease of <*C*_BP_>_SP_. Figure 11 shows that this is indeed the case: <*C*_BP_>_SP_ decreases with increasing OS volume fraction in the mobile phase, which is, in turn, mirrored by the accompanying decrease of <*C*_W_>_SP_ and increase of <*C*_OS_>_SP_. Because the decrease of <*C*_BP_>_SP_ mirrors the decrease of the experimental retention factor with increasing mobile-phase elution strength (cf. Figure 3), the value of <*C*_BP_>_SP_ can be taken as an indicator for the retentivity of the solvated stationary phase toward the analyte.

**Figure 11:**
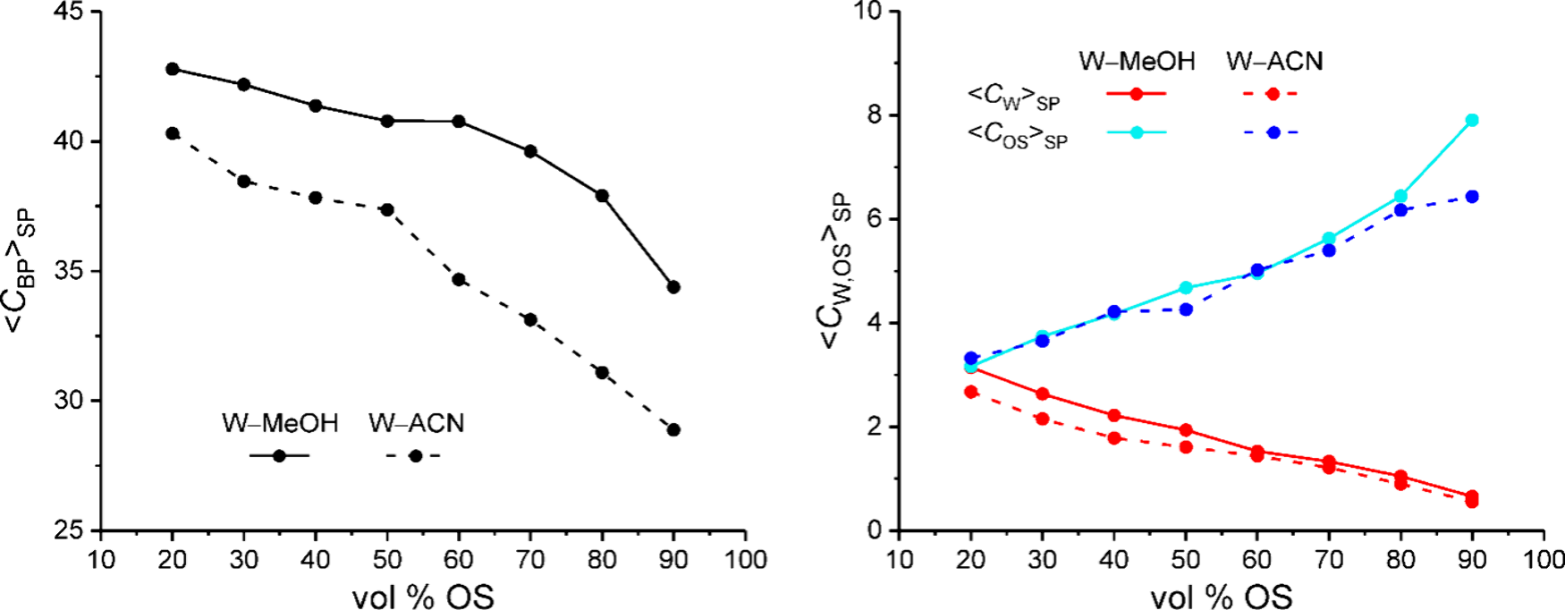
Evolution of the average analyte environment in the solvated stationary phase with increasing OS volume fraction in the W–MeOH or W–ACN mobile phase. The composition of the average analyte environment is quantified by the number of contacts with bonded-phase groups (left) and with W and OS molecules (right) per benzene molecule averaged over the extension of the solvated stationary phase (*z* ≤ *z*_SP_).

As already observed for the section-averaged <*C*_BP_> values in Figure 10, W–MeOH mobile phases generate higher <*C*_BP_>_SP_ values than W–ACN mobile phases at a given OS volume fraction. At the same time, <*C*_W_>_SP_ is not much higher with W–MeOH mobile phases, although the solvated stationary phase holds more W density with a W–MeOH than a W–ACN mobile phase at a given OS volume fraction (cf. Figures 4 and 5). Figure 11 shows no consistent correlation between the <*C*_BP_>_SP_ and <*C*_OS_>_SP_ values, eliminating the possibility that the increase of <*C*_OS_>_SP_ enables the decrease of <*C*_BP_>_SP_. Actually, it is the decrease of <*C*_BP_>_SP_ that entails the increase of <*C*_OS_>_SP_, as we will show next.

Figure 12 directly compares the benzene density distributions with W–MeOH vs W–ACN mobile phases at selected OS volume fractions and thus monitors the evolution of the benzene density distribution with increasing OS eluent strength as well as increasing OS volume fraction in the mobile phase. Higher OS eluent strength at a given OS volume fraction in the mobile phase shifts benzene density in the solvated stationary phase toward the bulk liquid region, first by shifting benzene density from the bonded-phase region (partitioning peak) to the interfacial region (adsorption peak), second by extending the adsorption peak toward the bulk liquid region, and third by redistributing benzene density within the interfacial region to larger *z*-values. Increasing the OS volume fraction in the mobile phase has principally the same effect on the benzene density distribution as increasing the OS eluent strength, but the achieved analyte density shift is smaller. Because *C*_BP_(*z*) decreases with increasing distance from the silica surface (cf. Figure 9), a benzene density shift toward the bulk liquid region corresponds to a decrease of <*C*_BP_>_SP_. Figure 12 shows that a benzene density shift toward the bulk liquid region, and thus a decrease of <*C*_BP_>_SP_, generally coincides with the retreat of W density from the interfacial region, as reflected by the decrease of <*C*_W_>_SP_ with increasing OS eluent strength and OS volume fraction in the mobile phase (cf. Figure 11). On the other hand, the OS density in the interfacial region increases with increasing OS volume fraction in the mobile phase, but decreases with increasing OS eluent strength. This shows that the benzene density distribution in the solvated stationary phase, and thus the value of <*C*_BP_>_SP_, responds to the W density, not to the OS density, in the interfacial region. The retreat of W molecules from the interfacial region allows benzene molecules to increasingly occupy analyte environments located closer to the bulk liquid region, which results in a decrease of <*C*_BP_>_SP_ and an increase of <*C*_OS_>_SP_, as analyte environments near the bulk liquid region have higher solvent density than environments located closer to the silica surface.

**Figure 12:**
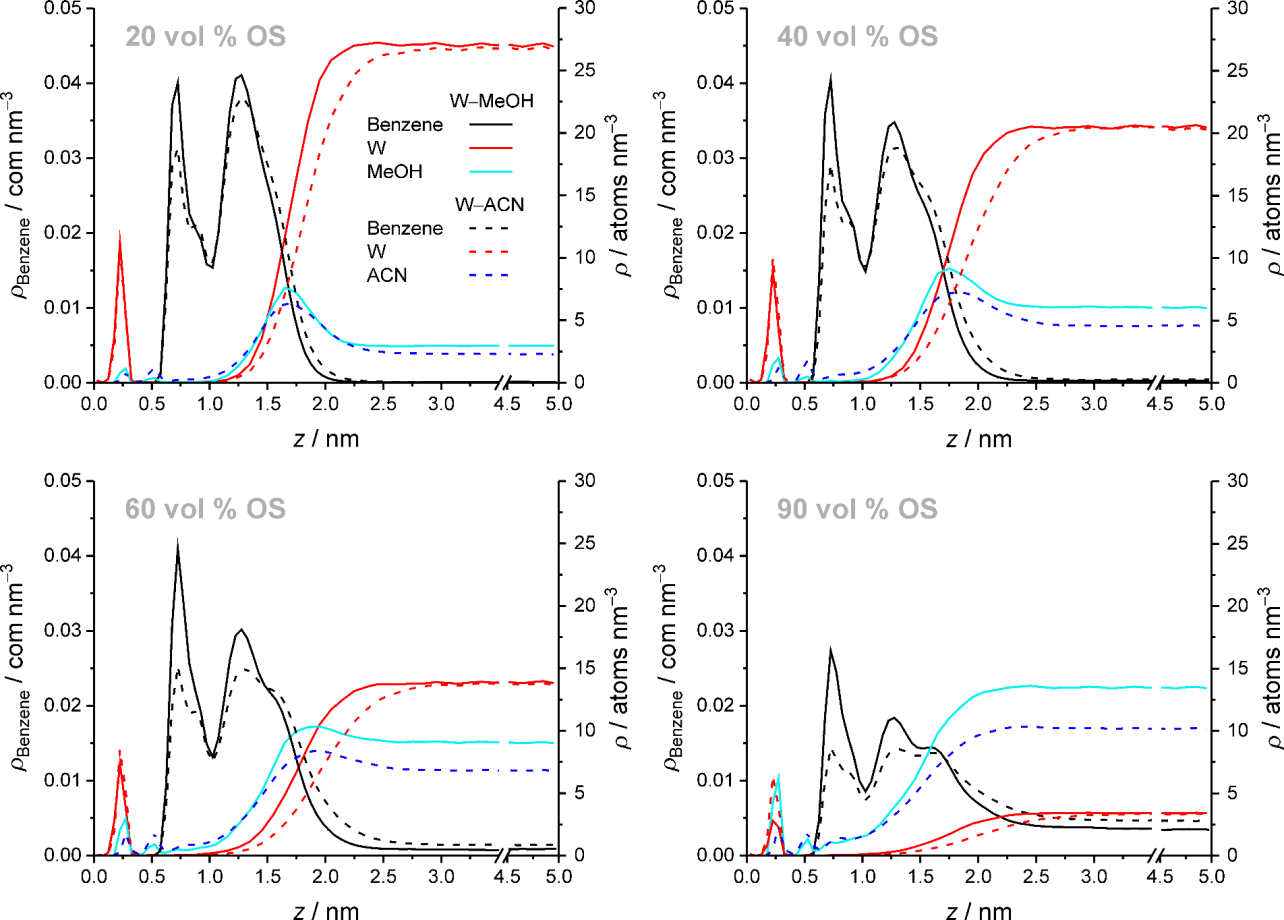
Response of the benzene density distribution to the mobile-phase composition at selected OS volume fractions in the W–OS mobile phase. Benzene density is redistributed and shifted to the bulk liquid region as W density recedes from the interfacial region with increasing mobile-phase elution strength.

Together, Figures 11 and 12 indicate that the population of the different analyte environments in the solvated stationary phase by benzene molecules is driven by the evasion of analyte–W contacts. This behavior reflects the preference of benzene molecules to largely exclude W molecules from their solvation shells (cf. Figure 2). As opposed to the bulk liquid, where solute molecules can shape their immediate environment to the extent admitted by W–OS hydrogen bonding, the composition of the immediate analyte environments available in the solvated stationary phase is decided upon the equilibration of the stationary phase with the running mobile phase. The preference of benzene molecules for different environments is then expressed by their population.

The benzene density profiles in Figure 8 had shown that the adsorption peak holds more benzene density than the partitioning peak regardless of the mobile-phase parameters. As explained in Section 3.3, small solutes, even flexible, apolar compounds, such as *n*-butane, generally prefer the interfacial region over the bonded-phase region, probably for entropic reasons, although the bonded-phase region offers low W presence.^15^ The retreat of W density from the interfacial region associated with increasing mobile-phase elution strength allows benzene molecules to leave the bonded-phase region and advance into the preferred interfacial region. This suggests that the benzene density in the interfacial region is limited by the local W density, as spatial restrictions for the analyte density do not exist at any location in the RPLC slit pore far beyond the employed analyte concentration.^62^

In summary, analyte retention decreases when the average number of bonded-phase contacts per analyte molecule within the stationary phase decreases, which occurs through an analyte density shift toward environments of lower bonded-phase density, enabled by the retreat of W density from the interfacial region with increasing mobile-phase elution strength. The retentivity of the stationary phase toward the analyte is therefore determined by the amount of W density that is dragged into the stationary phase during its solvation by the W–OS mobile phase.

## 4 Conclusions and Outlook

In the first part of our MD simulation study about the mobile-phase contribution to analyte retention and selectivity in RPLC, we have shown how the mobile phase controls analyte retention at the molecular level. The results finally provide a physicochemical foundation for the empirically derived mobile-phase elution strength, at least for the conditions represented by our study, which refer to the separation of small, neutral compounds on a silica-based, endcapped, C_18_ stationary phase with W–MeOH or W–ACN mobile phases. The molecular-level changes accompanying the macroscopic loss of analyte retention were investigated through monitoring the changes in the immediate analyte environments, whose composition was traced through spatially resolved contact analysis for benzene as the smallest, structurally simplest compound in our analyte ensemble. The immediate analyte environment reflects the local bonded-phase and solvent densities and thus varies gradually over the extension of the solvated stationary phase. The bonded-phase and solvent density distributions are, in turn, a function of the mobile-phase elution strength.

Increasing the mobile-phase elution strength generates higher OS density in the solvent-depleted bonded-phase region and lower W density in the interfacial region, enabling the redistribution and extension of bonded-phase and analyte density toward the bulk liquid region. Changes in the bonded-phase density distribution result from various conformational improvements of the C_18_ chains. Changes in the analyte density distribution, supported by the concomitant changes in the bonded-phase density distribution, result from the retreat of W density from the interfacial region, which allows the apolar benzene molecules to advance closer to the bulk liquid region and populate environments with lower bonded-phase density.

Regarding the average analyte environment in the solvated stationary phase, an analyte density shift toward the bulk liquid region corresponds to an overall loss of bonded-phase and W contacts. This means that the macroscopic loss of analyte retention with increasing mobile-phase elution strength is not solely motivated by the increasingly favorable solvation environment in the bulk liquid, which induces analyte molecules to leave the stationary phase, but also by decreasing W density in the interfacial region, which reduces the “grip” of the bonded phase on the analyte. The stationary phase-averaged number of bonded-phase contacts per analyte molecule decreases, like the retention factor, with increasing mobile-phase elution strength and can be regarded as a measure for the retentivity of the stationary phase toward the analyte.

What constitutes the elution strength of the W–OS mobile phase is the amount of W density that is dragged into the stationary phase and the extent of preferential analyte solvation permitted in the bulk liquid region. The mobile phase thus controls retention via the stationary-phase solvation and the analyte solvation shell, and we know that both depend on W–OS hydrogen bonding.^18,19^

Restricting our analysis in the first part of the study to the analyte benzene allowed us to eschew the consideration of preferential molecular orientation and solute–solvent hydrogen bonding. In the second part of the study,^42^ we investigate how the presence of side chains and hydrophilic functional groups that informs the solute polarity of the compounds in our ensemble, influences the population of the different analyte environments and ultimately the retention factor.

## Supplementary Material



## Data Availability

Input files for the MD simulations of this study are openly available in the Zenodo repository via the DOI: 10.5281/zenodo.15364716 under the license CC-BY-4.0 (Creative Commons Attribution 4.0 International).

## Notes

Number of W and OS molecules in the simulation box (Table S1); average W and OS densities and ratio of bonded-phase to solvent density in the solvated stationary phase (Table S2); contribution of backfolded and extended chains to the bonded-phase conformation (Tables S3–S6); and mobile-phase sensitivity of section-averaged benzene contacts (Figures S1–S3) (PDF)

## Abbreviations

ACN: acetonitrile
BP: bonded phase
bf: backfolded
C18: dimethyl-*n*-octadecylsilane
com: center-of-mass
ext: extended
HB: hydrogen bond
HILIC: hydrophilic interaction liquid chromatography
HPLC: high-performance liquid chromatography
MeOH: methanol
MD: molecular dynamics
OS: organic solvent
RDF: radial distribution function
RPLC: reversed-phase liquid chromatography
SP: stationary phase
W: water
