# Supplementary material for: Mobile-Phase Contributions to Analyte Retention and Selectivity in Reversed-Phase Liquid Chromatography: 1. General Effects
Source: J Phys Chem B. 2025 Jun 13;129(25):6385–400. doi: 10.1021/acs.jpcb.5c01695 (PMC12207586; doi:10.1021/acs.jpcb.5c01695)

Supporting Information

Mobile-Phase Contributions to Analyte Retention and Selectivity in Reversed-Phase Liquid Chromatography: 1. General Effects

Andreas Steinhoff, Alexandra Höltzel, and Ulrich Tallarek *

Department of Chemistry, Philipps-Universität Marburg, Hans-Meerwein-Strasse 4, 35032 Marburg, Germany

* Email: tallarek@staff.uni-marburg.de

Table S1. Number of W and OS molecules in the simulation box at a given OS volume fraction in the W–MeOH or W–ACN mobile phase.

	W–MeOH		W–ACN	
Vol % OS	N_{W}	N_{OS}	N_{W}	N_{OS}
10	33027	2444	32000	2199
20	29076	4254	28002	3713
30	25372	5980	23970	4902
40	21735	7669	20432	6227
50	18198	9318	16980	7440
60	14648	10923	13601	8568
70	11028	12529	10167	9741
80	7383	14098	6647	10842
90	3670	15591	3368	11832

Table S2. Average W and O densities and ratio of bonded-phase to solvent density in the solvated stationary phase at a given OS volume fraction in the W–MeOH or W–ACN mobile phase.

Vol % OS	W–MeOH			W–ACN		
	$\langle\rho_W\rangle^a$	$\langle\rho_{MeOH}\rangle^a$	$\langle\rho_{BP}/\rho_{solvent}\rangle^b$	$\langle\rho_W\rangle^a$	$\langle\rho_{ACN}\rangle^a$	$\langle\rho_{BP}/\rho_{solvent}\rangle^b$
	(atoms nm^{-3})			(atoms nm^{-3})		
10	11.31	1.59	1.19	10.22	1.56	1.30
20	9.57	2.35	1.29	7.89	2.29	1.51
30	8.13	3.00	1.38	6.53	2.76	1.65
40	6.88	3.58	1.47	5.36	3.24	1.78
50	5.70	4.14	1.56	4.44	3.59	1.91
60	4.60	4.64	1.66	3.51	3.90	2.07
70	3.52	5.13	1.77	2.68	4.20	2.23
80	2.39	5.64	1.91	1.75	4.46	2.47
90	1.22	6.13	2.09	0.92	4.67	2.74

[a] Densities were averaged over z = 0–2.525 nm. [b] BP density includes endcapping groups.

Table S3. Contribution of backfolded chains to the bonded-phase conformation with W–MeOH mobile phases.

	Backfolded, Tilted			Backfolded, Upright		
Vol % MeOH	Fraction (%)	z(CH_3-18) (nm)	N_{gauche}	Fraction (%)	z(CH_3-18) (nm)	N_{gauche}
10	9.6	0.72	3.92	16.7	0.73	4.18
20	7.9	0.73	3.93	17.3	0.73	4.19
30	7.5	0.73	3.90	17.1	0.73	4.20
40	7.2	0.73	3.94	15.9	0.73	4.19
50	7.7	0.73	3.93	16.3	0.73	4.24
60	6.4	0.73	3.97	17.0	0.73	4.26
70	6.8	0.73	3.94	16.0	0.73	4.19
80	6.1	0.73	3.93	15.8	0.74	4.22
90	5.8	0.73	3.92	15.9	0.74	4.25
average	7.2	0.73	3.93	16.4	0.73	4.21

Table S4. Contribution of backfolded chains to the bonded-phase conformation with W–ACN mobile phases.

	Backfolded, Tilted			Backfolded, Upright		
Vol % ACN	Fraction (%)	$z(CH_3\text{-}18)$ (nm)	N_{gauche}	Fraction (%)	$z(CH_3\text{-}18)$ (nm)	N_{gauche}
10	6.8	0.73	3.96	18.2	0.73	4.19
20	7.2	0.73	3.90	16.8	0.73	4.19
30	6.3	0.73	3.93	15.4	0.73	4.21
40	6.4	0.73	3.93	14.6	0.73	4.22
50	6.1	0.73	3.84	14.3	0.74	4.19
60	6.5	0.73	3.91	14.0	0.73	4.24
70	5.7	0.73	3.89	14.1	0.73	4.20
80	5.2	0.73	3.90	13.9	0.74	4.21
90	5.2	0.75	3.86	13.1	0.74	4.17
average	6.2	0.73	3.90	14.9	0.73	4.20

Table S5. Contribution of extended chains to the bonded-phase conformation with W–MeOH mobile phases.

	Extended, Tilted			Extended, Upright		
Vol % MeOH	Fraction (%)	$z(CH_3\text{-}18)$ (nm)	N_{gauche}	Fraction (%)	$z(CH_3\text{-}18)$ (nm)	N_{gauche}
10	25.6	1.34	3.73	48.2	1.34	3.98
20	22.7	1.36	3.71	52.2	1.36	3.99
30	21.0	1.37	3.72	54.5	1.37	3.97
40	24.1	1.39	3.67	52.8	1.40	3.98
50	23.8	1.42	3.69	52.2	1.42	3.96
60	20.5	1.43	3.70	56.1	1.44	3.98
70	21.9	1.44	3.68	55.3	1.46	3.94
80	21.6	1.48	3.66	56.6	1.49	3.96
90	20.8	1.51	3.62	57.5	1.53	3.94
average	20.2	1.42	3.67	53.9	1.42	3.97

Table S6. Contribution of extended chains to the bonded-phase conformation with W–ACN mobile phases.

	Extended, Tilted			Extended, Upright		
Vol % ACN	Fraction (%)	$z(CH_3\text{-}18)$ (nm)	N_{gauche}	Fraction (%)	$z(CH_3\text{-}18)$ (nm)	N_{gauche}
10	21.7	1.36	3.74	53.3	1.36	3.97
20	22.9	1.39	3.69	53.0	1.39	3.95
30	23.1	1.42	3.64	55.3	1.43	3.95
40	25.2	1.44	3.63	53.8	1.45	3.92
50	23.1	1.44	3.61	56.5	1.46	3.92
60	23.6	1.46	3.65	55.9	1.49	3.92
70	23.7	1.49	3.59	56.5	1.51	3.91
80	21.2	1.51	3.63	59.6	1.53	3.90
90	22.2	1.52	3.58	59.5	1.55	3.82
average	23.0	1.45	3.64	55.9	1.46	3.92

Figure S1. Sensitivity of the section-averaged number of bonded-phase contacts per benzene molecule to the mobile-phase composition. P1 and P2 indicate the silica-surface side and the bulk-liquid side of the partitioning peak, respectively, and A1 and A2 indicate the silica-surface side and the bulk-liquid side of the adsorption peak in the benzene density profiles (cf. Figure 8).

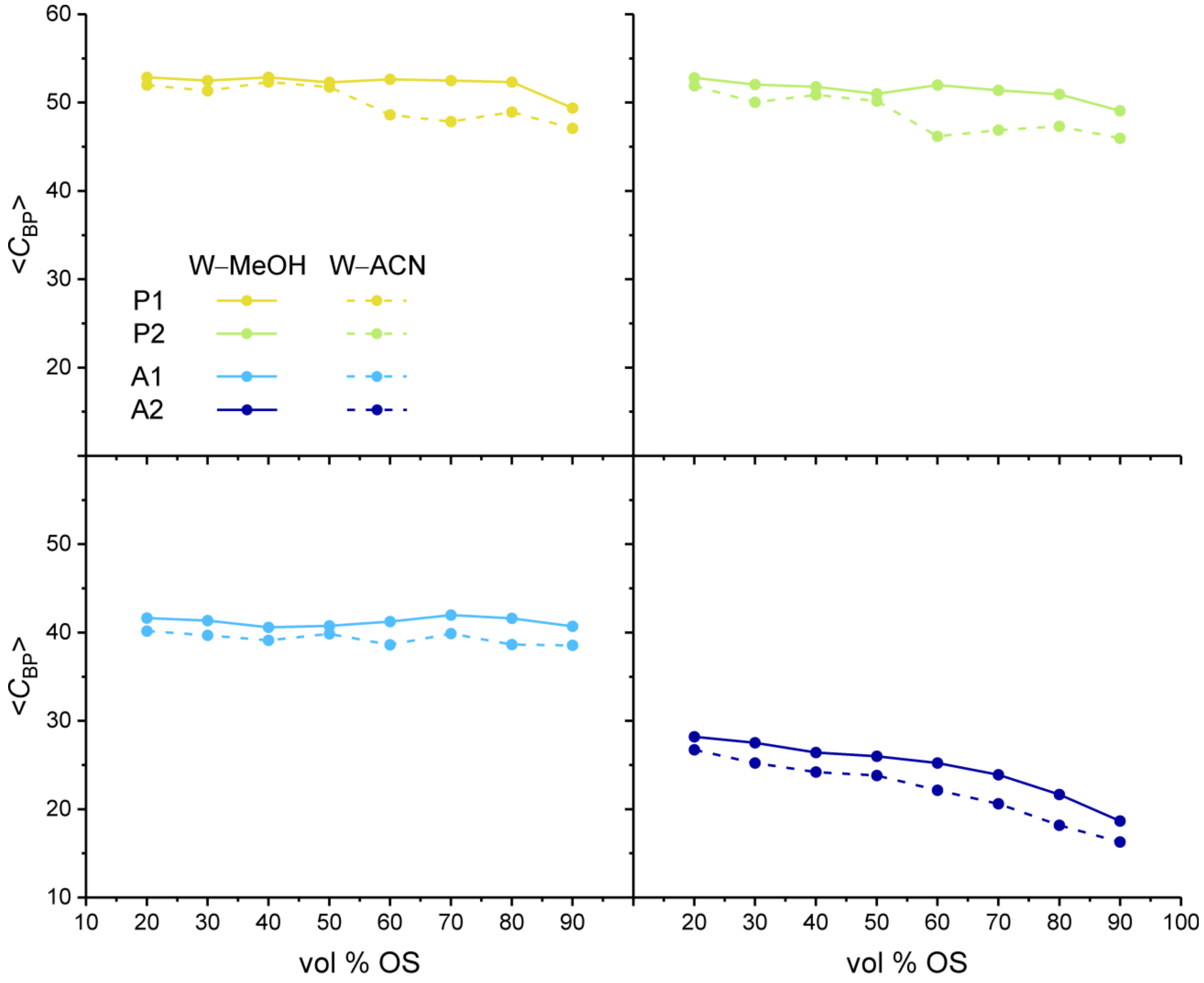

Figure S2. Sensitivity of the section-averaged number of W contacts per benzene molecule to the mobile-phase composition. Abbreviations as in Figure 8.

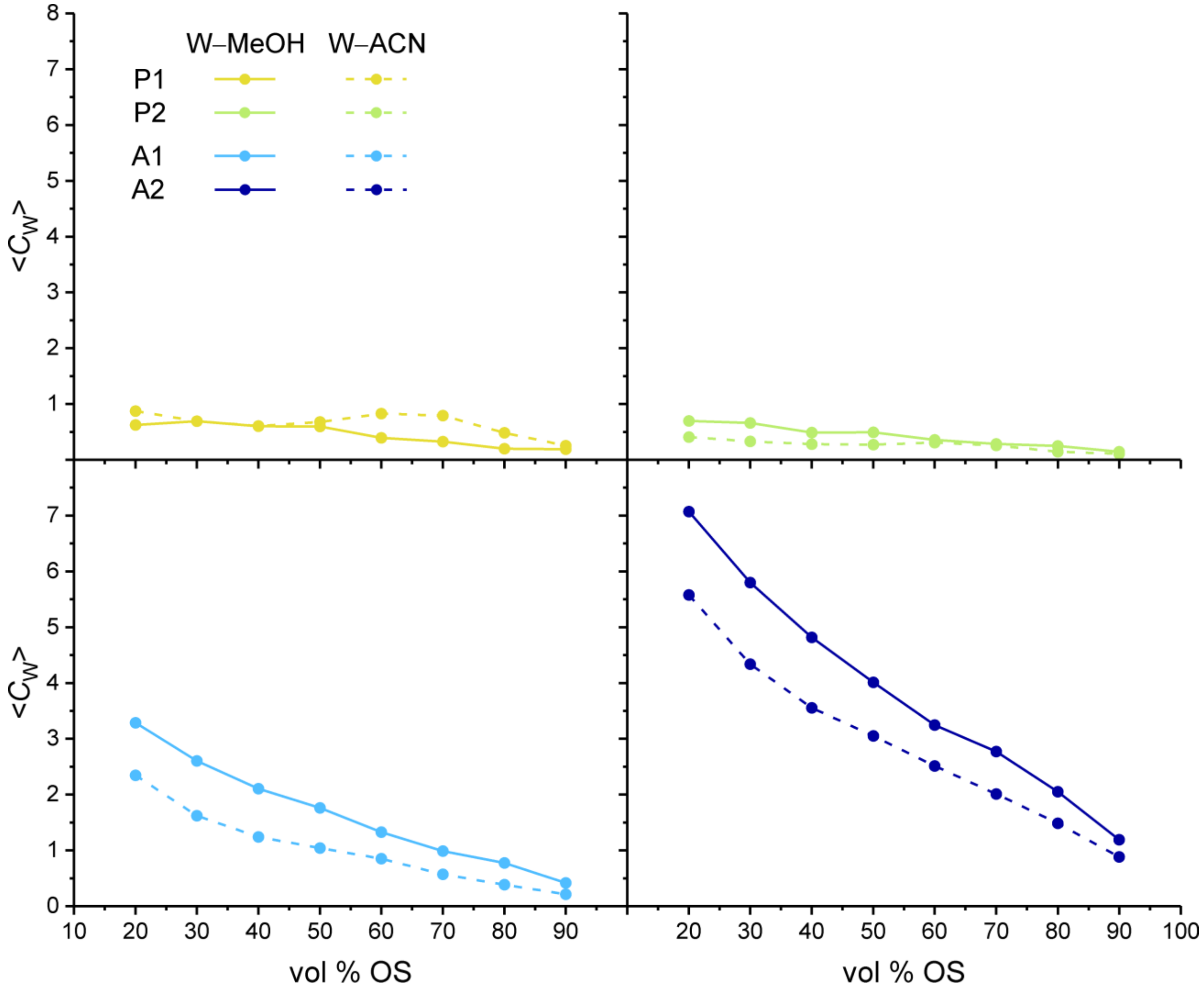

Figure S3. Sensitivity of the section-averaged number of OS contacts per benzene molecule to the mobile-phase composition. Abbreviations as in Figure 8.

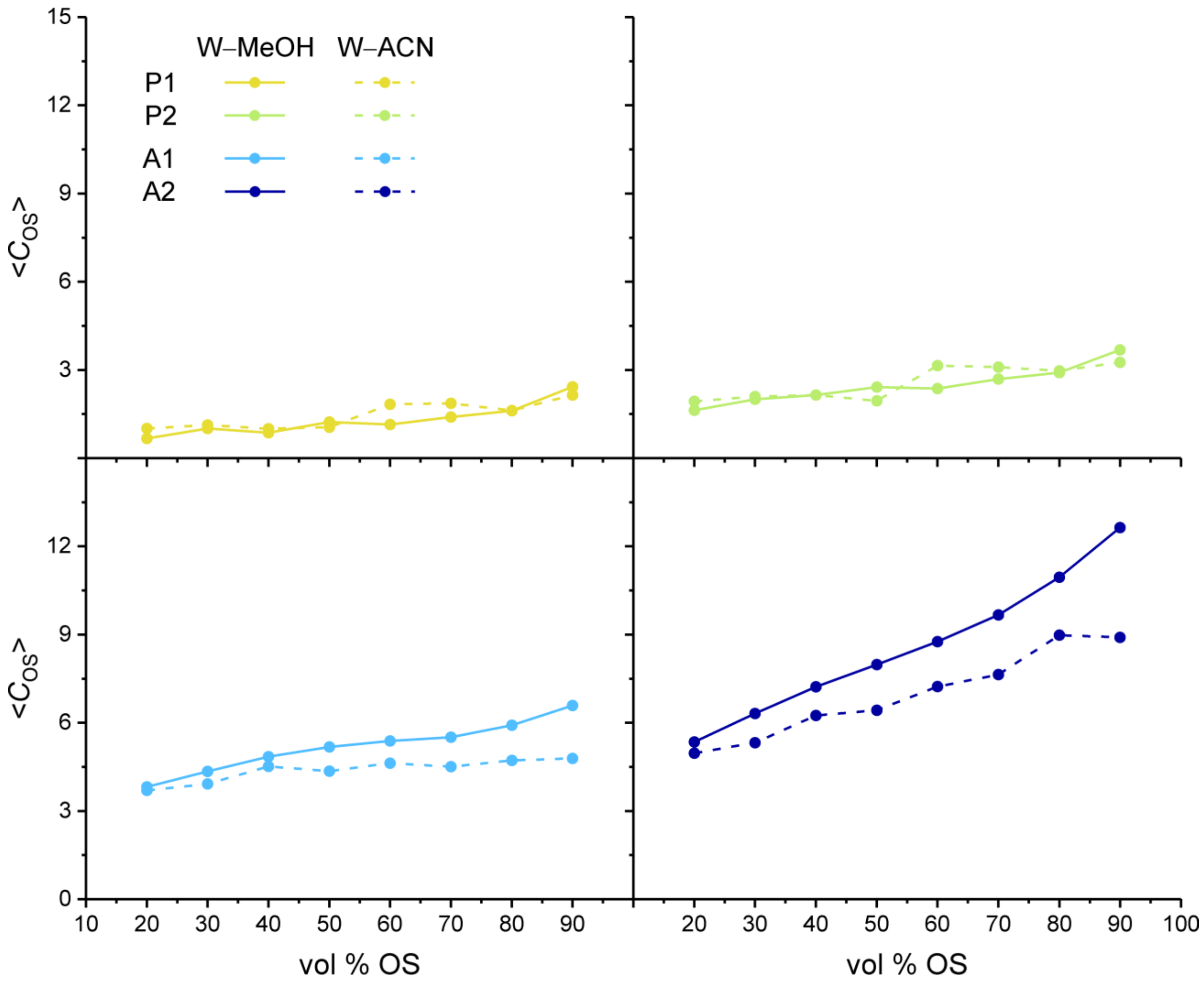

Supplement: Supplementary file 1 [file jp5c01695_si_001.pdf]
